# Validation of *in silico* biomarkers for drug screening through ordinal logistic regression

**DOI:** 10.3389/fphys.2022.1009647

**Published:** 2022-10-06

**Authors:** Da Un Jeong, Rakha Zharfarizqi Danadibrata, Aroli Marcellinus, Ki Moo Lim

**Affiliations:** ^1^ Computational Medicine Lab, Kumoh National Institute of Technology, Department of IT Convergence Engineering, Gumi, South Korea; ^2^ Computational Medicine Lab, Kumoh National Institute of Technology, Department of Medical IT Convergence Engineering, Gumi, South Korea

**Keywords:** torsades de pointes, proarrhythmic risk, drug toxicity, *in silico* biomarkers, ordinal logistic regression

## Abstract

Since the Comprehensive *in vitro* Proarrhythmia Assay (CiPA) initiation, many studies have suggested various *in silico* features based on ionic charges, action potentials (AP), or intracellular calcium (Ca) to assess proarrhythmic risk. These *in silico* features are computed through electrophysiological simulations using *in vitro* experimental datasets as input, therefore changing with the quality of *in vitro* experimental data; however, research to validate the robustness of *in silico* features for proarrhythmic risk assessment of drugs depending on *in vitro* datasets has not been conducted. This study aims to verify the availability of *in silico* features commonly used in assessing the cardiac toxicity of drugs through an ordinal logistic regression model and three *in vitro* datasets measured under different experimental environments and with different purposes. We performed *in silico* drug simulations using the Tomek-Ohara Rudy (ToR-ORD) ventricular myocyte model and computed 12 *in silico* features comprising six AP features, four Ca features, and two ion charge features, which reflected the effect and characteristics of each *in vitro* data for CiPA 28 drugs. We then compared the classific performances of ordinal logistic regressions according to these 12 *in silico* features and used *in vitro* datasets to validate which *in silico* feature is the best for assessing the proarrhythmic risk of drugs at high, intermediate, and low levels. All 12 *in silico* features helped determine high-risky torsadogenic drugs, regardless of the *in vitro* datasets used in the *in silico* simulation as input. In the three types of *in silico* features, AP features were the most reliable for determining the three Torsade de Pointes (TdP) risk standards. Among AP features, AP duration at 50% repolarization (APD_50_) was the best when individually using *in silico* features per *in vitro* dataset. In contrast, the AP repolarization velocity (dVm/dt_Max_repol_) was the best when merging all *in silico* features computed through three *in vitro* datasets.

## 1 Introduction

The S7B and E14 guidelines established by the International Council for Harmonization (ICH) are used during *in vitro* and *in vivo* drug safety assessments to determine the eventual development and distribution of discovered drug candidates. Many Torsade de Pointes (TdP)-inducible drugs have been successfully detected through these conventional guidelines with high sensitivity that inspect whether TdP is induced or not based on a human ether-à-go-go (hERG) blockage and QT prolongation (Cavero and Crumb, 2005; Shah, 2005; Sager et al., 2014). However, their low specificity has disrupted the development of new drugs, not only for negative-effect drugs but also for positive potential drugs (Colatsky et al., 2016; Fermini et al., 2016; Strauss et al., 2019). As a new paradigm to revise the current guidelines, the Comprehensive *in vitro* Proarrhythmia Assay (CiPA) was suggested at the Think Tank Meeting at the US Food and Drug Administration (FDA) headquarters in 2013 by 13 advanced medical institutions from seven countries (Sager et al., 2014). The CiPA comprises four components: the *in vitro* assessment of multiple human cardiac currents, *in silico* assessment of computer-reconstructed cellular models, *in vivo* electrocardiograph (ECG) assessment, and *in vitro* assessment using stem cell-derived ventricular cardiomyocytes.

Many studies have suggested various *in silico* features based on ionic charges, action potential (AP), and intracellular calcium (Ca) to assess proarrhythmic risk since the initiation of the CiPA (Mirams et al., 2011; Lancaster and Sobie, 2016). The ionic charge features qNet (Dutta et al., 2017) and qInward (Li et al., 2017), which are the amounts of charge in the Inet (I_NaL_, I_CaL_, I_Kr_, I_Ks_, I_K1_, and I_to_) and inward current (I_NaL_ and I_CaL_), respectively, showed an excellent ability to distinguish the proarrhythmic risk of drugs. Moreover, AP upstroke velocity (dVm/dt_Max_), peak AP (Vm_Peak_), AP duration at 90% repolarization (APD_90_), AP duration at 50% repolarization (APD_50_), APD triangulation (AP_tri_), and AP resting (Vm_rest_) are also commonly used as standard AP metrics to determine TdP risk as well as to predict electrophysiological instability (Mirams et al., 2011; Lancaster and Sobie, 2016). Similar to the AP metrics, Ca durations at 90% repolarization (CaD_90_), Ca durations at 50% repolarization (CaD_50_), Ca triangulation (CaD_tri_), and peak Ca (Ca_peak_) were extracted as Ca metrics from the intracellular Ca concentration trace (Lancaster and Sobie, 2016).

These *in silico* features are computed through electrophysiological simulations using *in vitro* experimental data as input, therefore changing based on the quality of *in vitro* experimental data; however, research to validate the robustness of *in silico* features for proarrhythmic risk assessment of drugs depends on *in vitro* datasets has not been conducted. This study aims to verify the availability of *in silico* features commonly used in assessing the cardiac toxicity of drugs through an ordinal logistic regression model and three *in vitro* datasets measured under different experimental environments and with different purposes. For this purpose, we computed 12 *in silico* features comprising six AP features, four Ca features, and two ion charge features through *in silico* drug simulation using three *in vitro* experimental datasets as input. Then, we compared the classification performances of ordinal logistic regression models according to these 12 *in silico* features and used three *in vitro* datasets to validate which *in silico* feature is the best for assessing the TdP risk of drugs at high, intermediate, and low levels.

## 2 Methods

### 2.1 Comprehensive *in vitro* proarrhythmia assay drug dataset

We used three CiPA experimental datasets from Li et al. Li et al. (2019), Chantest et al., and Nanion et al. Han et al. (2020), consisting of *in vitro* data for the same 28 drugs but with differences in their experimental conditions. The list of 28 CiPA drugs consisting of eight high-risk, eleven intermediate-risk, and nine low-risk drugs is in Table 1. Each dataset had an inhibition rate measured through a voltage clamp in seven ion channels of I_Na_, I_NaL_, I_Kr_, I_Ks_, I_K1_, I_to_, and I_CaL_ according to four concentration variations of the 28 CiPA drugs (https://github.com/FDA/CiPA/). All the datasets were preprocessed by following Crumb et al.’s methodology Crumb et al. (2016). First, the uncertainty of the *in vitro* dataset was quantified using the Markov chain Monte Carlo (MCMC) method proposed by Chang et al. (2017), generating 2,000 Hill curves within a 95% confidence interval. The half-maximal inhibitory concentration (IC_50_) and the slope coefficients at IC_50_ (Hill coefficients, h) were obtained from the 2,000 of Hill curves. These 2,000 IC50 and h values were used for *in silico* drug simulation as inputs to simulate the static binding of a drug for ion channels.

**TABLE 1:** List of the 28 drugs and their corresponding Cmax values.

Proarrhythmic risk level	Train drug		Test drug	
	Drug name	Cmax (nM)	Drug name	Cmax (nM)
High Risk	Quinidine	3,237	Disopyramide	742
	Sotalol	14,690	Ibutilide	100
	Dofetilide	2	Vandetanib	255
	Bepridil	33	Azimilide	70
Intermediate Risk	Cisapride	2.6	Clarithromycin	1,206
	Terfenadine	4	Clozapine	71
	Chlorpromazine	38	Domperidone	19
	Ondansetron	139	Droperidol	6.3
			Pimozide	0.43
			Astemizole	0.26
			Risperidone	1.81
Low Risk	Verapamil	81	Metoprolol	1,800
	Ranolazine	1,948.20	Nifedipine	7.7
	Diltiazem	122	Nitrendipine	3.02
	Mexiletine	4,129	Tamoxifen	21
			Loratadine	0.45

### 2.2 *In-silico* simulation and features

The *in silico* simulation was conducted using the Tomek-Ohara Rudy (ToR-ORd) model, a calibrated ORD ventricular myocyte electrophysiology model with the updated I_CaL_, I_Kr_, and Na^+^-Ca^2+^ exchangers to reproduce the depolarization, repolarization, and calcium dynamics of the AP trace and calcium transient (O’Hara et al., 2011; Tomek et al., 2019). The inhibited ionic current by the drug block was implemented by multiplying the drug-induced conductance block formulation instead of the original conductance as follows (Eqs. 1–3), Mirams et al., 2011):

$$I_{ion}^{\prime }=G_{ion}^{\prime }∙m_{ion}\left(V_{m}-E_{ion}\right)$$ (1)

$$G_{ion}^{\prime }=IF∙G_{ion}$$ (2)

$$inhibition\;factor\;\left(IF\right)=\frac{1}{1+{\left(\frac{\left[D\right]}{IC50}\right)}^{h}}$$ (3)

where $I_{ion}^{\prime }$ represents the remaining ionic currents after drug block, and $G_{ion}^{\prime }$ is the conductance block due to drug; V_m_ is the membrane potential; $G_{ion}$ , $m_{ion}$ , and $E_{ion}$ are the maximum conductance, gate variable, and equilibrium potential of the specific ionic current, respectively; and D is the drug concentration, which is set as 1-, 2-, 3-, and 4-fold the Cmax of a drug for the experimental uncertainty not becoming high (the Cmax value of each drug is listed in Table 1). All *in silico* drug simulations were performed under the steady-state condition of ventricular myocytes by saving the state values of the gates and currents after 10,000 beats without drug effect and inputting them as the initial values (Dutta et al., 2017). The AP shapes and corresponding ionic current profiles were generated by 1,000-stimulations at a 2,000 ms cycle length with a 0.1-ms time resolution; here, 2,000 ms of cycle length are 30 bpm of heart rate and mimic the bradycardia condition, where QT interval is prolonged and can be developed into TdP.

*In silico* features were calculated from the AP shapes and ionic current profiles when the repolarization velocity was maximal within the last 250 beats, which reached a steady state. Here, the beat in the maximal repolarization velocity reflects the worst situation for myocytes, such as the early after-depolarization. The extracted *in silico* features consisted of six AP features, four Ca features, and two ion charge features computed from each drug concentration. AP features were the velocities of the AP upstroke (dVm/dt_Max_) and AP repolarization (dVm/dt_Max_repol_), Vm_Peak_, APD_90_, APD_50_, and the difference between APD_90_ and APD_50_ (APD_tri_). The Ca features were Ca_Peak_, CaD_90_, CaD_50_, and the difference between CaD_90_ and CaD_50_ (CaD_tri_). The ion charge features were qNet and qInward.

As mentioned in Section 2.1, we bootstrapped *in vitro* experimental data and obtained 2,000 IC50 and h values for each drug from 2,000 Hill curves. *In silico* drug simulation used these 2,000 IC50 and h values as input to mimic the drug effect on ventricular myocyte, generating 2,000 AP shapes, Ca curves, and ionic curves per drug concentration. Since drug simulation was performed in four concentration conditions, which were Cmax×1, Cmax×2, Cmax×3, and Cmax×4, we generated 8,000 *in silico* biomarkers (2,000 IC50 and h values × four concentrations) for each drug. The average *in silico* features across the four drug concentrations were used for the input of the ordinal logistic regression model to assess the proarrhythmic risk, considering the balance between optimal risk stratification and reliable feature calculation, based on the TdP metric calculation method of Li et al. Li et al. (2019); that is, 2,000 *in silico* features were calculated per drug.

### 2.3 Model training and testing

The ordinal logistic regression model implemented using R was trained using 12 CiPA train drugs; the training drug set had 24,000 *in silico* features (12 drugs × averaging 2,000 *in silico* features across the four concentrations). Based on the distribution of *in silico* features in the training drug set, we decided on two threshold values for distinguishing high-, intermediate-, and low-risk drug toxicities. Threshold 1 identifies the low-risk and high/intermediate risk, and threshold 2 marks the high-risk and intermediate-risk/low (Li et al., 2019).

**FIGURE 1:**
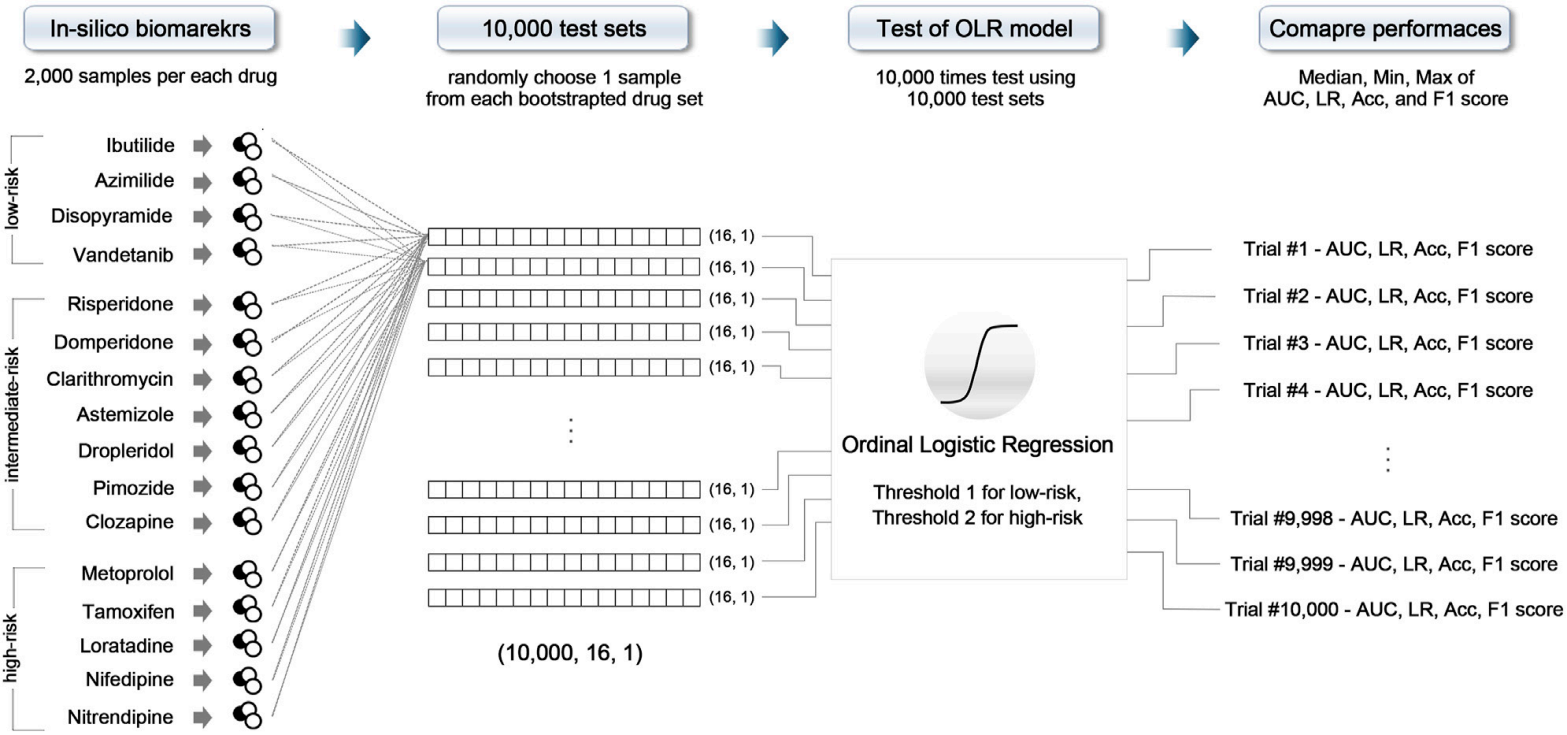
Schematic of the 10,000-repeated testing method; OLR, ordinal logistic regression; AUC, area under the receiver operating curve; LR, likelihood ratio; Acc, accuracy.

All models were validated using 16 CiPA test drugs through the 10,000-repeated testing method, as shown in Figure 1. The test drugs set consisted of 32,000 *in silico* features (16 drugs × averaging 2,000 *in silico* features across the four concentrations). First, we randomly chose one sample from 2,000 *in silico* features samples for each drug and then combined the samples to form one set; one test set consisted of 16 feature samples for 16 test drugs (one sample for each drug). We repeated this procedure 10,000 times, generating 10,000 test sets. Then, the model was evaluated 10,000 times using these 10,000 test sets (Li et al., 2019). As a result, we plotted 10,000 of the receiver operating curves (ROC) and compared the area under the curves (AUCs), likelihood ratio (LR), accuracy, and F1 score to evaluate the classification performance and classifier output quality.

$$Positive\;likelihood\;ratio\;\left(LR+\right)=\frac{sensitivity}{1-specificity}$$ (4)

$$Negative\;likelihood\;ratio\;\left(LR-\right)=\frac{1-sensitivity}{specificity}$$ (5)

$$Accuracy=\frac{TP+TN}{TP+TN+FN+FP}$$ (6)

$$F1\;score=2\frac{precision∙recall}{precision+recall}$$ (7)

Sensitivity (recall)=TP/(TP+FN) (8)

Specificity=TN/(TN+FP) (9)

Precision=TP/(TP+FP) (10)

where TP and TN are “true positives” and “true negatives,” which mean that the model correctly answers the actual positive/negative problems, respectively. Conversely, FP and FN are “false positives” and “false negatives,” which represent the mispredicted cases for the actual negative/positive problem as positive/negative.

## 3 Results

We performed an F-test and two-sample t-tests to validate the *in silico* biomarkers computed through drug simulations using the ToR-ORd model depends on the *in vitro* experimental dataset as independent sets. Summaries of the *in silico* biomarkers computed through the drug simulations per *in vitro* experimental dataset can be found in Supplementary Table S1. The F-test showed that the variance of each dataset was different; accordingly, the two-sample t-test was performed by assuming unequal variance. All biomarkers differed significantly according to the *in vitro* datasets (*p*-value < 0.001, Supplementary Table S2).

Through the logistic regression model, we determined thresholds for assessing the TdP-risk depending on the *in vitro* datasets, including a combined set of the three datasets. According to the *in silico* features for distinguishing the three levels of the proarrhythmic risk, two threshold values of ordinal logistic regression models are shown in Table 2. Threshold 1 is the boundary value to sort those with low risk from those with high/intermediate risk, while threshold 2 separates those with high risk from intermediate/low risk. The difference between thresholds 1 and 2 in the AP and ionic charge features was the largest in the Li dataset and the smallest in the Nanion dataset. For the Ca features, the difference between thresholds 1 and 2 was the largest in the Li dataset but smallest in the Chantest dataset. In the Nanion dataset, CaD_90_ was unsuitable for assessing the proarrhythmic risk of the drugs when the ordinal logistic regression was used. The distribution of features in all the datasets and the corresponding thresholds are shown in Supplementary Figures S1–S16.

**TABLE 2:** Thresholds of 12 *in silico* biomarkers for classifying TdP risk. Threshold 1 (TH1) was used to distinguish the low-risk from the intermediate/high-risk groups, while threshold 2 (TH2) was used to distinguish the high-risk from the low/intermediate-risk groups. Merged, the combined set containing all three datasets.

*In-silico* features	Li et al.		Chantest et al.		Nanion et al.		Merged	
	TH1	TH2	TH1	TH2	TH1	TH2	TH1	TH2
dVm/dt_Max_repol_ (mV)	−0.382	−0.287	−0.3994	−0.3697	−0.450	−0.471	−0.414	−0.370
dVm/dt_Max_ (mV)	297.1	285.3	294.6	291.3	296.9	293.9	296.3	289.2
Vm_Peak_ (mV)	21.6	22.7	22	22.3	21.7	22.1	21.7	22.3
APD_90_ (ms)	341.3	394.4	335.8	351.7	314.1	302.9	329.3	355.3
APD_50_ (ms)	293.5	338.8	288.7	301.7	271.1	261.4	283.3	305.2
APD_tri_ (ms)	47.8	55.6	47.1	50	42.9	41.5	45.9	49.9
Ca_Peak_ (nM)	0.00025	0.00027	0.000269	0.000279	0.000270	0.000275	0.000263	0.000276
CaD_90_ (ms)	592.5	604.4	579.1	574.9	−	−	581.4	581.0
CaD_50_ (ms)	284.3	296.5	278.1	277.4	272.2	267.9	277.9	281.7
CaD_tri_ (ms)	309.4	303.9	301.0	297.8	300.7	296.6	303.5	299.2
qNet	0.128	0.071	0.132	0.113	0.147	0.150	0.139	0.108
qInward	0.938	1.041	0.936	0.977	0.934	0.960	0.932	0.996

**FIGURE 2:**
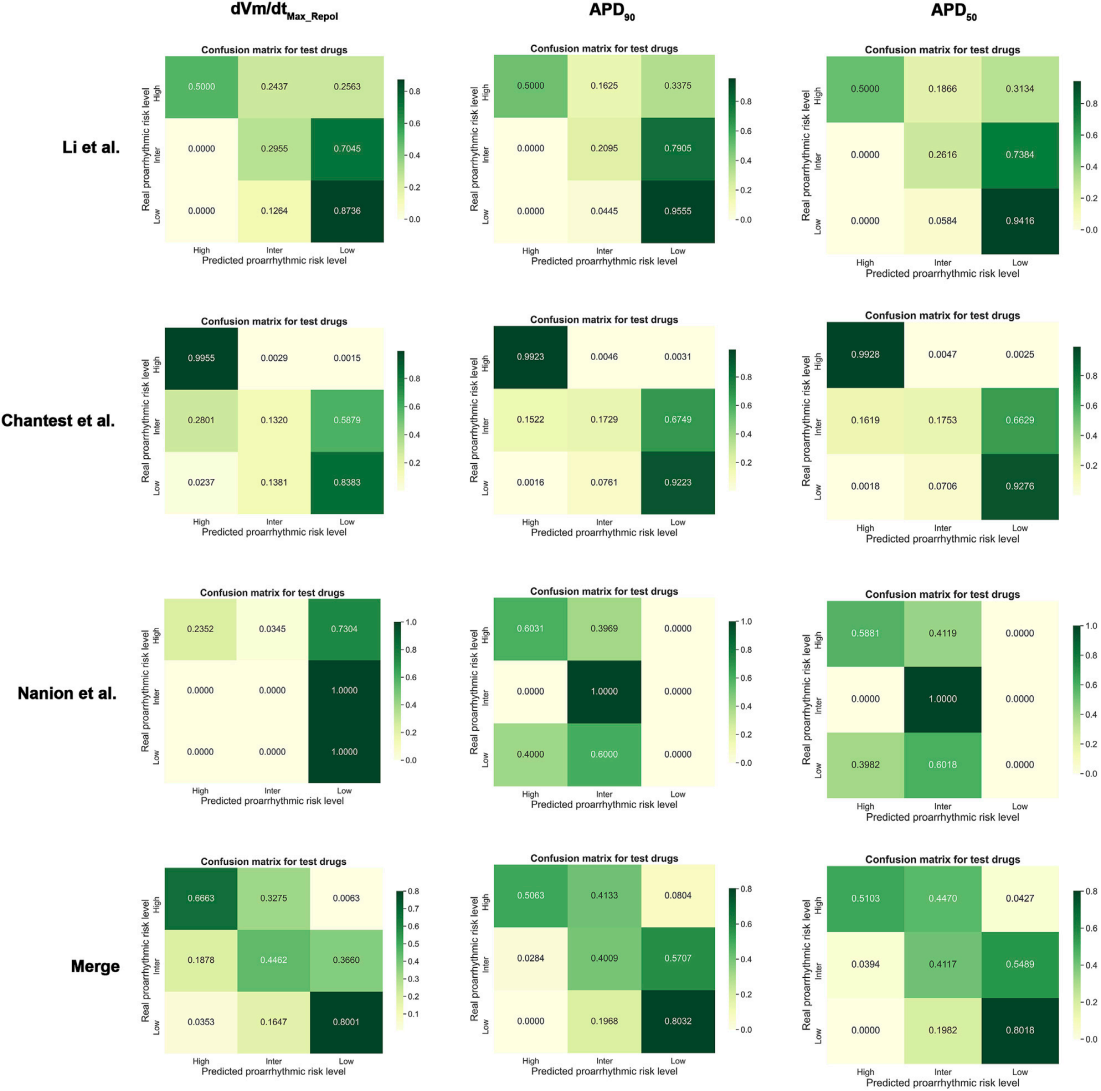
Normalized confusion matrices for dVm/dt_Max_repol_, APD_90_, and APD_50_; Merged, the combined set containing all three datasets.

Summaries of the ordinal logistic regression model are shown in Tables 3–5 and Supplementary Tables S3–S5. AP features appeared to be more helpful in classifying high-risk drugs than other risk levels (Table 3 and Supplementary Table S3). Especially, the classification accuracies for the high-risk levels of the Chantest dataset were over 0.90 AUCs when using dVm/dt_Max_repol_, APD_90_, APD_50_, and APD_tri_; the LRs of these features were also within excellent ranges (LR+> 10, LR-<0.1) and the F1 scores were over 0.80. The dVm/dt_Max_repol_, APD_90_, APD_50_, and APD_tri_ were moderate, above 0.70 AUCs for classifying high-risk drugs in the Li dataset and the merged set of the three datasets and for classifying intermediate-risk drugs in the Nanion dataset. However, in the Nanion dataset, the AP features were ineffective for classifying between low- and high-risk. When individually assessing the TdP risk using each dataset, comparing not only the performances of the ordinal logistic regression models but also the LRs, the features APD_50_ and APD_90_ showed the best performance in the Li and Chantest datasets, while APD_tri_ showed the best performance in the Nanion dataset. However, when using the merged dataset, dVm/dt_Max_repol_ was the best feature for assessing TdP risk (Figure 2).

**TABLE 3:** Summary of classification performance using action potential (AP) features; classification performances according to the AP features shows the median, minimal and maximal values after evaluating the model through the 10,000-test algorithm; AUC, the area under the receiver operating curves; Merged, the combined set containing all three datasets; ACC, accuracy; One asterisk (*) denotes the intermediate performance over 0.7 of median values, and two asterisks (**) denote the excellent performance over 0.8 of median values.

AP feature	Dataset	Acc	AUC			F1-score		
			Low	Intermediate	High	Low	Inter	High
dVm/dt_Max_repol_	Li.	0.68	0.67 (0.54–0.82)	0.53 (0.33–0.73)	0.75* (0.75–0.75)	0.57 (0.47–0.71)	0.36 (0.00–0.67)	0.67 (0.67–0.67)
	Chantest	0.71*	0.72* (0.63–0.86)	0.52 (0.39–0.66)	0.92** (0.75–0.96)	0.62 (0.53–0.77)	0.22 (0.00–0.55)	0.80** (0.60–0.89)
	Nanion	0.71*	0.50 (0.50–0.50)	0.72* (0.61–0.78)	0.67 (0.42–0.79)	0.00 (0.00–0.00)	0.74* (0.67–0.78)	0.50 (0.00–0.67)
	Merged	0.74*	0.76* (0.67–0.95)	0.60 (0.33–0.87)	0.79* (0.58–1.00)	0.67 (0.57–0.91)	0.50 (0.00–0.86)	0.67 (0.40–1.00)
dVm/dt_Max_	Li.	0.59	0.58 (0.29–0.76)	0.52 (0.28–0.80)	0.58 (0.50–0.83)	0.50 (0.25–0.67)	0.22 (0.00–0.77)	0.33 (0.25–0.75)
	Chantest	0.65	0.63 (0.53–0.77)	0.44 (0.28–0.59)	0.83** (0.50–0.96)	0.53 (0.43–0.67)	0.00 (0.00–0.40)	0.75* (0.25–0.89)
	Nanion	0.58	0.55 (0.50–0.64)	0.50 (0.33–0.50)	0.62 (0.50–0.75)	0.50 (0.48–0.56)	0.00 (0.00–0.00)	0.40 (0.00–0.67)
	Merged	0.58	0.54 (0.29–0.72)	0.48 (0.28–0.71)	0.58 (0.46–0.83)	0.47 (0.25–0.62)	0.20 (0.00–0.62)	0.33 (0.22–0.75)
Vm_Peak_	Li.	0.64	0.52 (0.38–0.71)	0.56 (0.44–0.76)	0.62 (0.62–0.62)	0.36 (0.29–0.60)	0.53 (0.40–0.75)	0.40 (0.40–0.40)
	Chantest	0.75*	0.70* (0.42–0.90)	0.62 (0.35–0.82)	0.79* (0.54–0.96)	0.57 (0.20–0.89)	0.57 (0.17–0.80)	0.67 (0.36–0.89)
	Nanion	0.5	0.65 (0.56–0.80)	0.35 (0.28–0.59)	0.29 (0.21–0.54)	0.50 (0.40–0.75)	0.17 (0.00–0.40)	0.15 (0.13–0.40)
	Merged	0.55	0.47 (0.38–0.71)	0.42 (0.29–0.63)	0.54 (0.50–0.71)	0.33 (0.29–0.60)	0.31 (0.15–0.63)	0.29 (0.25–0.57)
APD_90_	Li.	0.68	0.68 (0.49–0.82)	0.57 (0.33–0.71)	0.75 (0.75–0.75)	0.59 (0.44–0.71)	0.25 (0.00–0.60)	0.67 (0.67–0.67)
	Chantest	0.74*	0.77* (0.63–0.82)	0.57 (0.39–0.64)	0.96** (0.79–0.96)	0.67 (0.53–0.71)	0.25 (0.00–0.44)	0.89** (0.67–0.89)
	Nanion	0.73*	0.50 (0.50–0.50)	0.72* (0.61–0.78)	0.67 (0.42–0.79)	0.00 (0.00–0.00)	0.74* (0.67–0.78)	0.50 (0.00–0.67)
	Merged	0.70*	0.72* (0.58–0.86)	0.55 (0.33–0.75)	0.75* (0.67–0.88)	0.62 (0.50–0.77)	0.46 (0.00–0.71)	0.67 (0.50–0.86)
APD_50_	Li.	0.69	0.68 (0.49–0.82)	0.59 (0.39–0.79)	0.75* (0.75–0.75)	0.59 (0.44–0.71)	0.40 (0.00–0.73)	0.67 (0.67–0.67)
	Chantest	0.74*	0.77* (0.63–0.82)	0.57 (0.39–0.64)	0.96** (0.79–0.96)	0.67 (0.53–0.71)	0.25 (0.00–0.44)	0.89** (0.67–0.89)
	Nanion	0.72*	0.50 (0.50–0.50)	0.72* (0.56–0.78)	0.67 (0.42–0.83)	0.00 (0.00–0.00)	0.74* (0.64–0.78)	0.50 (0.00–0.75)
	Merged	0.71*	0.72* (0.63–0.86)	0.55 (0.33–0.80)	0.75* (0.67–0.88)	0.62 (0.53–0.77)	0.46 (0.00–0.77)	0.67 (0.50–0.86)
APD_tri_	Li.	0.66	0.64 (0.49–0.73)	0.57 (0.39–0.64)	0.75* (0.75–0.75)	0.56 (0.44–0.62)	0.25 (0.00–0.44)	0.67 (0.67–0.67)
	Chantest	0.72*	0.67 (0.58–0.82)	0.52 (0.39–0.64)	0.96** (0.79–0.96)	0.57 (0.50–0.71)	0.22 (0.00–0.44)	0.89** (0.67–0.89)
	Nanion	0.70*	0.50 (0.50–0.50)	0.71* (0.52–0.83)	0.75* (0.46–0.79)	0.00 (0.00–0.00)	0.71* (0.53–0.82)	0.60 (0.22–0.67)
	Merged	0.66	0.63 (0.49–0.82)	0.53 (0.33–0.71)	0.75* (0.71–0.88)	0.53 (0.44–0.71)	0.36 (0.00–0.60)	0.67 (0.57–0.86)

**TABLE 4:** Summary of classification performance using calcium (Ca) features; classification performances according to the AP features shows the median, minimal and maximal values after evaluating the model through the 10,000-test algorithm; AUC, the area under the receiver operating curves; Merged, the combined set containing all three datasets; ACC, accuracy; One asterisk (*) denotes the intermediate performance over 0.7 of median values, and two asterisks (**) denote the excellent performance over 0.8 of median values.

Ca feature	Dataset	ACC	AUC			F1-score		
			Low	Inter	High	Low	Inter	High
Ca_Peak_	Li.	0.53	0.61 (0.37–0.65)	0.42 (0.17–0.73)	0.42 (0.17–0.62)	0.44 (0.18–0.50)	0.31 (0.00–0.67)	0.20 (0.00–0.44)
	Chantest	0.67	0.65 (0.42–0.75)	0.56 (0.35–0.82)	0.71* (0.38–0.83)	0.50 (0.20–0.67)	0.53 (0.17–0.80)	0.55 (0.18–0.75)
	Nanion	0.53	0.75* (0.56–0.80)	0.40 (0.28–0.59)	0.33 (0.25–0.58)	0.67 (0.40–0.75)	0.18 (0.00–0.46)	0.17 (0.14–0.43)
	Merged	0.51	0.43 (0.34–0.57)	0.48 (0.28–0.66)	0.38 (0.25–0.67)	0.31 (0.27–0.46)	0.33 (0.00–0.57)	0.00 (0.00–0.50)
CaD_90_	Li.	0.54	0.44 (0.44–0.58)	0.50 (0.39–0.57)	0.62 (0.62–0.79)	0.37 (0.37–0.50)	0.00 (0.00–0.25)	0.44 (0.44–0.67)
	Chantest	0.39	0.37 (0.27–0.62)	0.50 (0.33–0.66)	0.12 (0.08–0.29)	0.18 (0.00–0.50)	0.00 (0.00–0.55)	0.00 (0.00–0.15)
	Nanion	-	-	-	-	-	-	-
	Merged	0.46	0.43 (0.28–0.56)	0.50 (0.39–0.71)	0.33 (0.12–0.46)	0.31 (0.15–0.40)	0.00 (0.00–0.60)	0.17 (0.00–0.31)
CaD_50_	Li.	0.55	0.44 (0.44–0.63)	0.39 (0.28–0.52)	0.71* (0.71–0.71)	0.37 (0.37–0.53)	0.00 (0.00–0.22)	0.57 (0.57–0.57)
	Chantest	0.39	0.37 (0.18–0.52)	0.50 (0.39–0.64)	0.12 (0.04–0.33)	0.18 (0.00–0.36)	0.00 (0.00–0.44)	0.00 (0.00–0.17)
	Nanion	0.78*	0.75* (0.61–0.80)	0.71* (0.52–0.83)	0.75* (0.50–0.88)	0.67 (0.44–0.75)	0.71* (0.56–0.82)	0.67 (0.00–0.86)
	Merged	0.61	0.57 (0.38–0.76)	0.52 (0.33–0.79)	0.75* (0.58–0.92)	0.46 (0.29–0.67)	0.22 (0.00–0.73)	0.60 (0.40–0.80)
CaD_tri_	Li.	0.51	0.56 (0.37–0.61)	0.52 (0.33–0.66)	0.33 (0.12–0.62)	0.40 (0.18–0.44)	0.22 (0.00–0.55)	0.17 (0.00–0.46)
	Chantest	0.43	0.47 (0.33–0.71)	0.44 (0.22–0.71)	0.17 (0.08–0.58)	0.31 (0.17–0.60)	0.00 (0.00–0.60)	0.00 (0.00–0.43)
	Nanion	0.53	0.71* (0.52–0.80)	0.40 (0.28–0.60)	0.33 (0.25–0.58)	0.60 (0.36–0.75)	0.18 (0.00–0.50)	0.17 (0.14–0.43)
	Merged	0.48	0.47 (0.38–0.62)	0.44 (0.22–0.71)	0.42 (0.21–0.62)	0.33 (0.29–0.50)	0.18 (0.00–0.60)	0.20 (0.00–0.44)

**TABLE 5:** Summary of classification performance using ion charge features; classification performances according to the AP features shows the median, minimal and maximal values after evaluating the model through the 10,000-test algorithm; AUC, the area under the receiver operating curves; Merged, the combined set containing all three datasets; ACC, accuracy; One asterisk (*) denotes the intermediate performance over 0.7 of median values, and two asterisks (**) denote the excellent performance over 0.8 of median values.

Ion charge feature	Dataset	Acc	AUC			F1-score		
			Low	Inter	High	Low	Inter	High
qNet	Li.	0.7*	0.67 (0.58–0.82)	0.55 (0.48–0.75)	0.75 (0.75–0.75)	0.57 (0.50–0.73)	0.46 (0.33–0.71)	0.67 (0.67–0.67)
	Chantest	0.73*	0.76* (0.67–0.91)	0.52 (0.39–0.79)	0.92** (0.71–0.96)	0.67 (0.57–0.83)	0.22 (0.00–0.73)	0.80** (0.55–0.89)
	Nanion	0.57	0.44 (0.29–0.48)	0.50 (0.33–0.57)	0.75* (0.38–0.79)	0.37 (0.25–0.40)	0.00 (0.00–0.25)	0.60 (0.00–0.67)
	Merged	0.72*	0.71* (0.61–0.90)	0.56 (0.35–0.87)	0.71* (0.62–0.88)	0.60 (0.44–0.89)	0.53 (0.17–0.86)	0.57 (0.44–0.86)
qInward	Li.	0.73*	0.70* (0.55–0.70)	0.65 (0.45–0.78)	0.71* (0.62–0.88)	0.57 (0.29–0.57)	0.67 (0.47–0.78)	0.57 (0.44–0.86)
	Chantest	0.57	0.70* (0.50–0.70)	0.44 (0.33–0.64)	0.62 (0.46–0.71)	0.57 (0.00–0.57)	0.00 (0.00–0.44)	0.47 (0.35–0.53)
	Nanion	0.43	0.51 (0.46–0.70)	0.39 (0.28–0.50)	0.21 (0.17–0.58)	0.25 (0.22–0.57)	0.00 (0.00–0.00)	0.13 (0.12–0.44)
	Merged	0.65	0.65 (0.55–0.70)	0.59 (0.28–0.79)	0.71* (0.50–0.83)	0.50 (0.29–0.57)	0.40 (0.00–0.73)	0.53 (0.38–0.67)

The performance of the ordinal logistic regression model using Ca features was generally worse than when using AP features (Table 4 and Supplementary Table S4). Ca features aside from CaD_90_ were moderate for classifying the TdP-risk in the Nanion dataset only. CaD_90_ was not distinguished into the three TdP-risk levels of drugs using an ordinal logistic regression model. However, CaD_50_ can classify drugs into the three TdP-risk groups primarily in the Nanion dataset, with a tolerable accuracy of over 0.70 AUCs; the LR+ and LR- of the low- and high-risk groups were satisfied to be minimally acceptable (LR+> 2, LR-<0.5). The F1 scores were only good for those with intermediate risk as 0.71 but moderate for those with low- and high-risk at both 0.67. For the Chantest dataset, Ca_peak_ could only be used to determine the high-risk drugs, with 0.71 AUC and LRs only satisfying the minimum acceptable conditions. Similarly, CaD_50_ showed functional potential for assessing only the high-risk with 0.71 AUC for the Li dataset and 0.75 AUC for the merged dataset, respectively. The LRs of high-risk drugs in the merged dataset reached the minimum acceptable levels (LR+ = 3.0 and LR- = 0.33), while in the Li dataset, only LR + reached 6.0, satisfying a minimum acceptable condition; the LR- value was 1.47.

Table 5 and Supplementary Table S5 show summaries of the classification performance using the two ionic charge features of qNet and qinward. Generally, qNet seemed more helpful than qInward in determining the TdP-risk levels of the *in silico* features computed using the ToR-ORD model. The performance using qNet was excellent for classifying the high-risk group in the Chantest dataset, with 0.92 AUC (F1 score = 0.80); good for classifying low risk in the Chantest dataset, with 0.76 AUC (F1 score = 0.67) and high-risk in the Li dataset, with 0.75 AUC (F1 score = 0.67). In the Nanion dataset, the performance using qNet was similar to the dVm/dt_Max_repol_ when using the AP features; even though qNet could only potentially classify high risk, with 0.75 AUC and the minimum acceptable LRs, qInward was unsuitable for assessing the TdP-risk in the Nanion dataset. Accordingly, qNet sorted the *in silico* features of the merged datasets into low- and high-risk, both at 0.71 AUCs.

## 4 Discussion

This study validated 12 *in silico* features computed using the ToR-ORD model to assess the proarrhythmic risk based on ordinal logistic regression. These *in silico* features were generated from drug simulations using three different *in vitro* experimental datasets. The classification performance from ordinal logistic regression using each feature was compared to find the best *in silico* features to assess the torsadogenic risk of drugs. The main findings of this study are as follows:

1. All 12 *in silico* features computed through the ToR-ORD model help determine the high-risky torsadogenic drugs, regardless of the *in vitro* datasets used.
2. In the three types of *in silico* features, AP features were the most reliable for determning the three TdP-risk standards.
3. Among AP features, APD_50_ was the best to determine the three TdP-risk standards when individually using *in silico* features per *in vitro* dataset without merging them. In contrast, when merging three datasets, the dVm/dt_Max_repol_ is the best feature.

The Li and Chantest datasets showed similar aspects in classifying the TdP-risk using ordinal logistic regression according to the *in silico* features. We believe that this was because the *in vitro* experimental dataset of Chantest et al. was obtained by strictly following the methodology of Li et al. Han et al. (2020). Both datasets showed better performance results for dVm/dt_Max_repol_ than for dVm/dt_Max_ (Table 2 and Figure 2). We guess this was because of the limited time resolution set in the writing steps of the AP trace in the *in silico* simulation; we set the time resolution for calculation as 0.1 ms considering the computation efficiency. In the AP generation phase, dVm/dt_Max_ is the upstroke velocity of the zero step, and dVm/dt_Max_repol_ denotes the repolarization velocity of the third step (Shih, 1994; Grunnet, 2010). The spike and dome morphology of the upstroke phase can be easily lost depending on the time resolution compared to the repolarization morphology. That is, as dVm/dt_Max_ gets affected more directly by the time resolution compared to dVm/dt_Max_repol_, the time resolution we set in this study may not be sufficient to consider the difference in the upstroke morphology between proarrhythmic drugs. Despite the insufficient time resolution, dVm/dt_Max_ could classify the high-risk drugs in the Chantest dataset, which means that the time resolution of the *in silico* simulation also needs to be calibrated along with the observed dataset.

Unlike the Li and Chantest datasets, the distribution of most *in silico* features computed from the Nanion *in vitro* dataset was too unstable to sort the proarrhythmic risk and did not show satisfactory classification performance to assess the TdP-risk. We hypothesized that this was because the ranges of the *in silico* features in the low-risk and high-risk drugs overlapped widely (Supplementary Figures S9–S12). For example, in the training drugs, the dVm/dt_Max_repol_ values of diltiazem, mexiletine, and ranolazine, which are low-risk, are distributed in the high-risk ranges, causing the threshold 1 value for low-risk to be fitted only for verapamil. Furthermore, the dVm/dt_Max_repol_ values in the test drugs were also in the high-risk range, and the maximal value of the test drugs was -0.4507, smaller than the threshold 1 value of -0.450. Accordingly, the AUC for the low-risk drugs in the Nanion dataset was all 0.50, from the first quarter to the third quarter (Supplementary Figure S9). In the Nanion dataset, the results for dVm/dt_Max_repol_ corresponded to the likelihood ratio. The LR + for high-risk drugs had a minimum acceptance level of 2, which means that if the dVm/dt_Max_repol_ values of a drug are over -0.471, the threshold 2 value, the drug is more likely to be high-risk. However, the LR- for high-risk drugs was not satisfied with a minimum acceptance level of 0.5, which means that even though the threshold 2 value does not classify a drug as high-risk, it can indeed be high-risk (Aggarwal and Ranganathan, 2018).

The calcium features in this study could not reflect the drug effect in determining the proarrhythmic risk based on the poor performance in the original logistic regression in all datasets. Only CaD_50_ showed potential to classify high-risk drugs, denoting that the repolarization time in transient calcium might capture the relevant information due to the high TdP-risk. Most *in silico* features computed from the Nanion dataset did not have sufficient classification performance to assess the proarrhythmic risk; however, only calcium features seemed to be fit for classification; in particular, CaD_50_ can be used to categorize the three TdP-risk levels in the Nanion dataset. We speculate that this was due to the *in silico* results computed from the Nanion dataset, which have remarkable differences in the transient calcium current compared to other ionic currents in the proarrhythmic drugs. Indeed, the Nanion dataset was obtained not only by following the methodology of Li et al., but also by specializing in ion channel pharmacology for calcium and sodium channels (Han et al., 2020). This result supports the idea that *in silico* features calculated through drug simulations can have electrophysiological differences along with *in vitro* datasets.

The CiPA research groups suggested using an ordinal logistic regression model using the qNet value calculated based on the hERG assay through the inhibition rate of six to seven ionic currents that are mainly changed by proarrhythmic drugs. They reported excellent AUCs of 0.90 and 0.98 for classifying the low- and high-risk drugs, respectively, based on the qNet thresholds (Crumb et al., 2016; Li et al., 2019). Furthermore, APD_90_, APD_50_, and diastolic Ca also showed good performances at 0.84, 0.85, and 0.85 for low risk drugs and 0.98, 0.99, and 0.99 for high risk drugs (Li et al., 2019). This study also calculated the TdP metric values by strictly following their methodology, but the classification performances here based on the these *in silico* feature thresholds were lower than theirs. This may be due to the difference between *in silico* cardiac cell models and the fact that the qNet calculated from the Inet of the ToR-ORD model using the reformulated I_CaL_, I_Kr_, and Na^+^-Ca^2+^ exchanger reflected the experimental dataset, not the original ORD model (Tomek et al., 2019). Indeed, the qNet values of the Nanion dataset have opposite aspects according to the TdP-risk compared to the Li and Chantest datasets, where we observed that a higher qNet was more dangerous to the TdP in the training drugs (Supplementary Figure S12A). Accordingly, in the Nanion dataset, the value of qNet threshold 1 was smaller than threshold 2.

The ORD model is an *in silico* ventricular cell model commonly used in drug research that was optimized by Dutta et al. to observe cell responses corresponding to the drug blocks (Dutta et al., 2017). The ToR-ORD model used in this study revised the formulations of the I_CaL_, I_Kr_, and Na^+^-Ca^2+^ exchanger to make ionic balances during the repolarization time, which can more realistically mimic experimental/clinical data than the original ORD model (Tomek et al., 2019). This study used three experimental datasets that were measured under different conditions and purposes to validate the 12 *in silico* features. Therefore, we chose the ToR-ORD model instead of the optimized ORD model to reflect the characteristics of each experimental dataset.

The classification performances were not super high, even APD_50_ or dVm/dt_Max_repol_, which though were the best, depending on changes of the ventricular myocyte model and *in vitro* experimental data used for *in silico* simulation (Table 2 and Figure 2). We think that classifying drug safety using just one TdP feature is not sufficient to cover the variations of *in silico* model and *in vitro* data. In future studies, we will find the best model to assess drug safety by considering multiple *in silico* parameters simultaneously and using advanced machine learning techniques such as deep learning.

As a limitation of this study, we performed drug simulations without calibrating the *in silico* cardiac cell model corresponding to the experimental datasets. Previous studies have suggested various calibration algorithms to determine the proarrhythmic risk of drugs (Øvstebø et al., 2003; Carter et al., 2018; Tomek et al., 2019; Han et al., 2020). The calibration methodology that considers environment variables or individual physiological characteristics of used ionic channels depending on the experimental datasets helps determine TdP risk. This study focused on validating the robustness of *in silico* features to determine the TdP-risk according to the experimental datasets obtained under different experimental environments and with different purposes, as mentioned above. Furthermore, a standard ordinal logistic regression model must be defined using a criterion dataset before calibration. However, we did not decide on one as the standard because all three *in vitro* datasets used in this study were open-source. Therefore, we assumed that the classification performance of some features may improve if their thresholds were calibrated, but this does not strongly affect the main findings of this study.

## Data Availability

The datasets presented in this study can be found in online repositories. The names of the repository/repositories and accession number(s) can be found in the article/Supplementary Material.

## References

[B1] Aggarwal R. Ranganathan P. (2018). Understanding diagnostic tests - Part 3: Receiver operating characteristic curves. Perspect. Clin. Res. 9, 145–148. 10.4103/picr.PICR_87_18 30090714 PMC6058507

[B2] Carter J. A. Barros A. I. Nóbrega J. A. Donati G. L. (2018). Traditional calibration methods in atomic spectrometry and new calibration strategies for inductively coupled plasma mass spectrometry. Front. Chem. 6, 504–525. 10.3389/fchem.2018.00504 30483492 PMC6242947

[B3] Cavero I. Crumb W. (2005). ICH S7B draft guideline on the non-clinical strategy for testing delayed cardiac repolarisation risk of drugs: A critical analysis. Expert Opin. Drug Saf. 4, 509–530. 10.1517/14740338.4.3.509 15934857

[B4] Chang K. C. Dutta S. Mirams G. R. Beattie K. A. Sheng J. Tran P. N. (2017). Uncertainty quantification reveals the importance of data variability and experimental design considerations for *in silico* proarrhythmia risk assessment. Front. Physiol. 8, 917–17. 10.3389/fphys.2017.00917 29209226 PMC5702340

[B5] Colatsky T. Fermini B. Gintant G. Pierson J. B. Sager P. Sekino Y. (2016). The comprehensive *in vitro* proarrhythmia assay (CiPA) initiative — update on progress. J. Pharmacol. Toxicol. Methods 81, 15–20. 10.1016/j.vascn.2016.06.002 27282641

[B6] Crumb W. J. Vicente J. Johannesen L. Strauss D. G. (2016). An evaluation of 30 clinical drugs against the comprehensive *in vitro* proarrhythmia assay (CiPA) proposed ion channel panel. J. Pharmacol. Toxicol. Methods 81, 251–262. 10.1016/j.vascn.2016.03.009 27060526

[B7] Dutta S. Chang K. C. Beattie K. A. Sheng J. Tran P. N. Wu W. W. (2017). Optimization of an *in silico* cardiac cell model for proarrhythmia risk assessment. Front. Physiol. 8, 1–15. 10.3389/fphys.2017.00616 28878692 PMC5572155

[B8] Fermini B. Hancox J. C. Abi-Gerges N. Bridgland-Taylor M. Chaudhary K. W. Colatsky T. (2016). A new perspective in the field of cardiac safety testing through the comprehensive *in vitro* proarrhythmia assay paradigm. J. Biomol. Screen. 21, 1–11. 10.1177/1087057115594589 26170255

[B9] Grunnet M. (2010). Repolarization of the cardiac action potential. Dose an increase in repolarization capacity constitute a new anti-arrhythmic principle? Acta Physiol. (Oxf). 198, 1–48. 10.1111/j.1748-1716.2009.02072.x 20132149

[B10] Han X. Samieegohar M. Ridder B. J. Wu W. W. Randolph A. Tran P. (2020). A general procedure to select calibration drugs for lab-specific validation and calibration of proarrhythmia risk prediction models: An illustrative example using the CiPA model. J. Pharmacol. Toxicol. Methods 105, 106890. 10.1016/j.vascn.2020.106890 32574700

[B11] Lancaster M. C. Sobie E. A. (2016). Improved prediction of drug-induced Torsades de Pointes through simulations of dynamics and machine learning algorithms. Clin. Pharmacol. Ther. 00, 371–379. 10.1002/cpt.367 PMC6375298 26950176

[B12] Li Z. Dutta S. Sheng J. Tran P. N. Wu W. Chang K. (2017). Improving the *in silico* assessment of proarrhythmia risk by combining hERG (Human Ether-à-go-go-Related Gene) channel-drug binding kinetics and multichannel pharmacology. Circ. Arrhythm. Electrophysiol. 10, e004628–e004640. 10.1161/CIRCEP.116.004628 28202629

[B13] Li Z. Ridder B. J. Han X. Wu W. W. Sheng J. Tran P. N. (2019). Assessment of an in silico mechanistic model for proarrhythmia risk prediction under the CiPA initiative. Clin. Pharmacol. Ther. 105, 466–475. 10.1002/cpt.1184 30151907 PMC6492074

[B14] Mirams G. R. Cui Y. Sher A. Fink M. Cooper J. Heath B. M. (2011). Simulation of multiple ion channel block provides improved early prediction of compounds' clinical torsadogenic risk. Cardiovasc. Res. 91, 53–61. 10.1093/cvr/cvr044 21300721 PMC3112019

[B15] O’Hara T. Virág L. Varró A. Rudy Y. (2011). Simulation of the undiseased human cardiac ventricular action potential: Model formulation and experimental validation. PLoS Comput. Biol. 7, e1002061–e1002090. 10.1371/journal.pcbi.1002061 21637795 PMC3102752

[B16] Øvstebø R. Haug K. B. F. Lande K. Kierulf P. (2003). PCR-based calibration curves for studies of quantitative gene expression in human monocytes: Development and evaluation. Clin. Chem. 49, 425–432. 10.1373/49.3.425 12600954

[B17] Sager P. T. Gintant G. Turner J. R. Pettit S. Stockbridge N. (2014). Rechanneling the cardiac proarrhythmia safety paradigm: A meeting report from the cardiac safety research consortium. Am. Heart J. 167, 292–300. 10.1016/j.ahj.2013.11.004 24576511

[B18] Shah R. R. (2005). Drugs, QTc interval prolongation and final ICH E14 guideline: An important milestone with challenges ahead. Drug Saf. 28, 1009–1028. 10.2165/00002018-200528110-00003 16231954

[B19] Shih H.-T. (1994). Anatomy of the action potential in the heart. Tex. Heart Inst. J. 21, 30–41. Available at: https://www.ncbi.nlm.nih.gov/pmc/articles/PMC325129/pdf/thij00036-0042.pdf. 7514060 PMC325129

[B20] Strauss D. G. Gintant G. Li Z. Wu W. Blinova K. Vicente J. (2019). Comprehensive *in vitro* proarrhythmia assay (CiPA) update from a cardiac safety research consortium / health and environmental sciences Institute / FDA meeting. Ther. Innov. Regul. Sci. 53, 519–525. 10.1177/2168479018795117 30157676

[B21] Tomek J. Bueno-Orovio A. Passini E. Zhou X. Minchole A. Britton O. (2019). Development, calibration, and validation of a novel human ventricular myocyte model in health, disease, and drug block. Elife 8, e48890–e48938. 10.7554/eLife.48890 31868580 PMC6970534

