# Supplementary material for: Validation of in silico biomarkers for drug screening through ordinal logistic regression
Source: Front Physiol. 2022 Oct 6;13:1009647. doi: 10.3389/fphys.2022.1009647 (PMC9583152; doi:10.3389/fphys.2022.1009647)
Supplement: Supplementary file 1 [file DataSheet1.PDF]

Supplementary Material

Validation of In-silico Biomarkers for Drug Screening through Logistic Regression

Da Un Jeong[1], Rakha Zharfarizqi Danadibrata[1], Aroli Marcellinus[1], Ki Moo Lim[1, 2*]

*** Correspondence:** Ki Moo Lim: kmlim@kumoh.ac.kr

1 Supplementary Figure and Table

1.1 Supplementary Figures

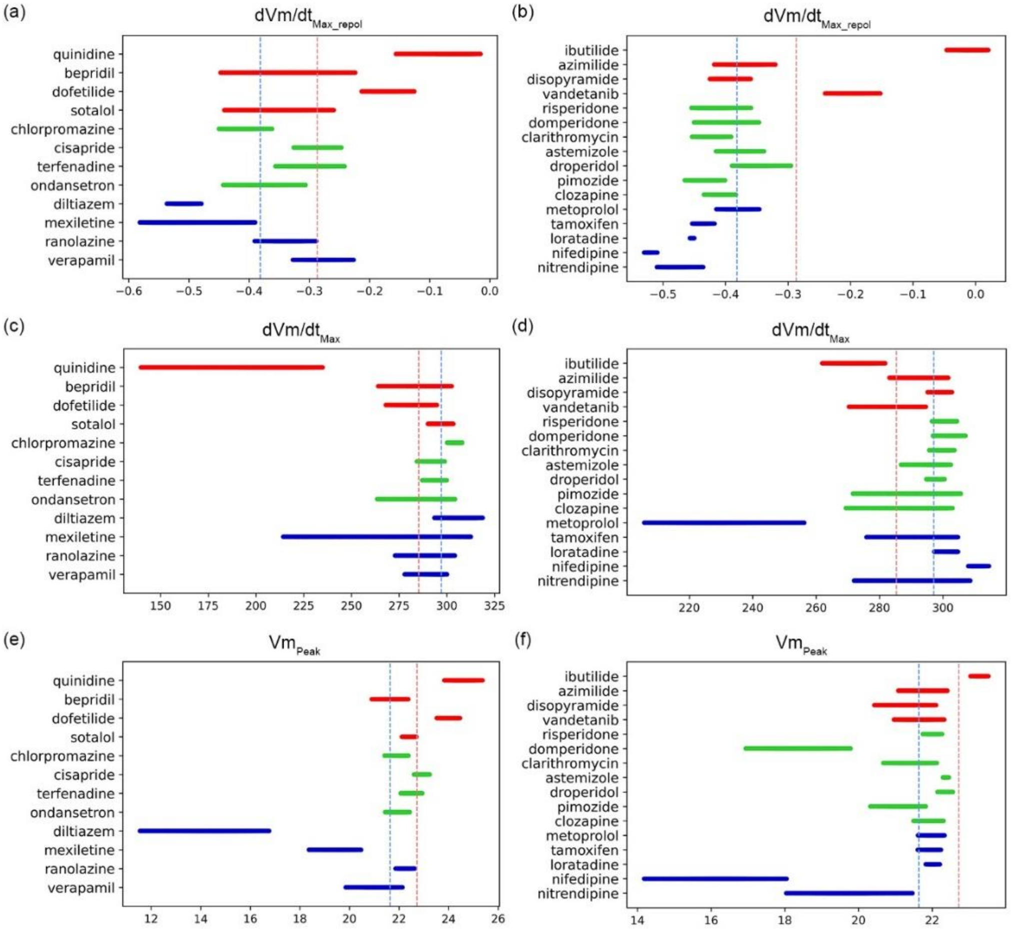

Supplementary Figure S 1. Distributions of dVm/dt_{Max_repol}, dVm/dt_{Max}, and Vm_{Peak} as *in-silico* AP features in the Li dataset; (a, c, e), for 12 train drugs; (b, d, f), for 16 test drugs; red, green, and blue horizontal lines denote the distribution of high, intermediate, and low-risk drugs; the blue dashed line is threshold 1 for distinguishing the low-risk drug from the high/intermediate-risk; the red dashed line is threshold 2 for determining the high-risk drug from the intermediate/low-risk.

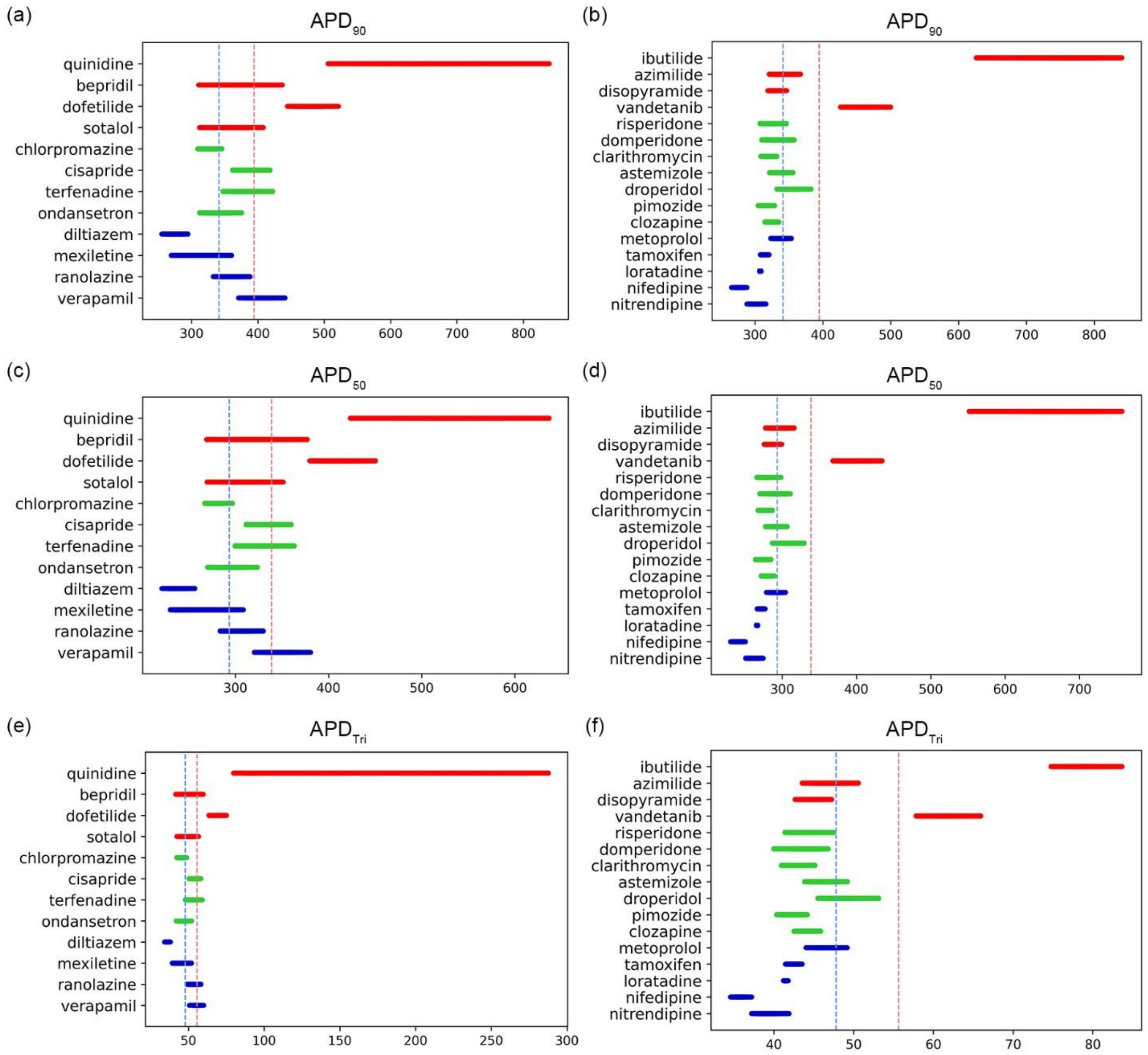

Supplementary Figure S 2. Distributions of APD_{90}, APD_{50}, and APD_{tri} as *in-silico* AP features in the Li dataset; (a, c, e), for 12 train drugs; (b, d, f), for 16 test drugs; red, green, and blue horizontal lines denote the distribution of high, intermediate, and low-risk drugs; the blue dashed line is threshold 1 for distinguishing the low-risk drug from the high/intermediate-risk; the red dashed line is threshold 2 for determining the high-risk drug from the intermediate/low-risk.

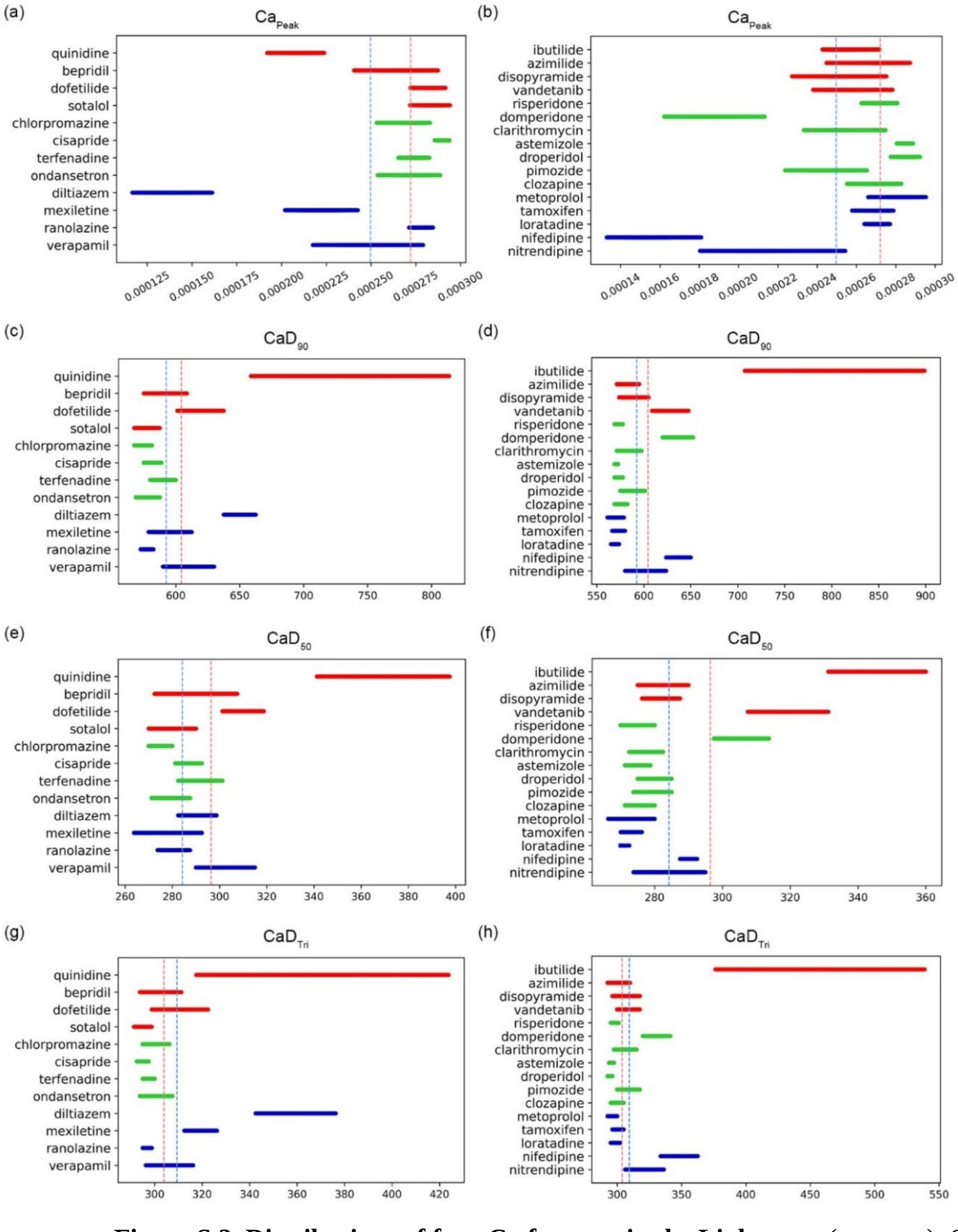

Supplementary Figure S 3. Distributions of four Ca features in the Li dataset; (a, c, e, g), Ca_{Peak}, CaD_{90}, CaD_{50}, and CaD_{tri} of 12 train drugs; (b, d, f, h), Ca_{Peak}, CaD_{90}, CaD_{50}, and CaD_{tri} of 16 test drugs; red, green, and blue horizontal lines denote the distribution of high, intermediate, and low-risk drugs; the blue dashed line is threshold 1 for distinguishing the low-risk drug from the high/intermediate-risk; the red dashed line is threshold 2 for determining the high-risk drug from the intermediate/low-risk.

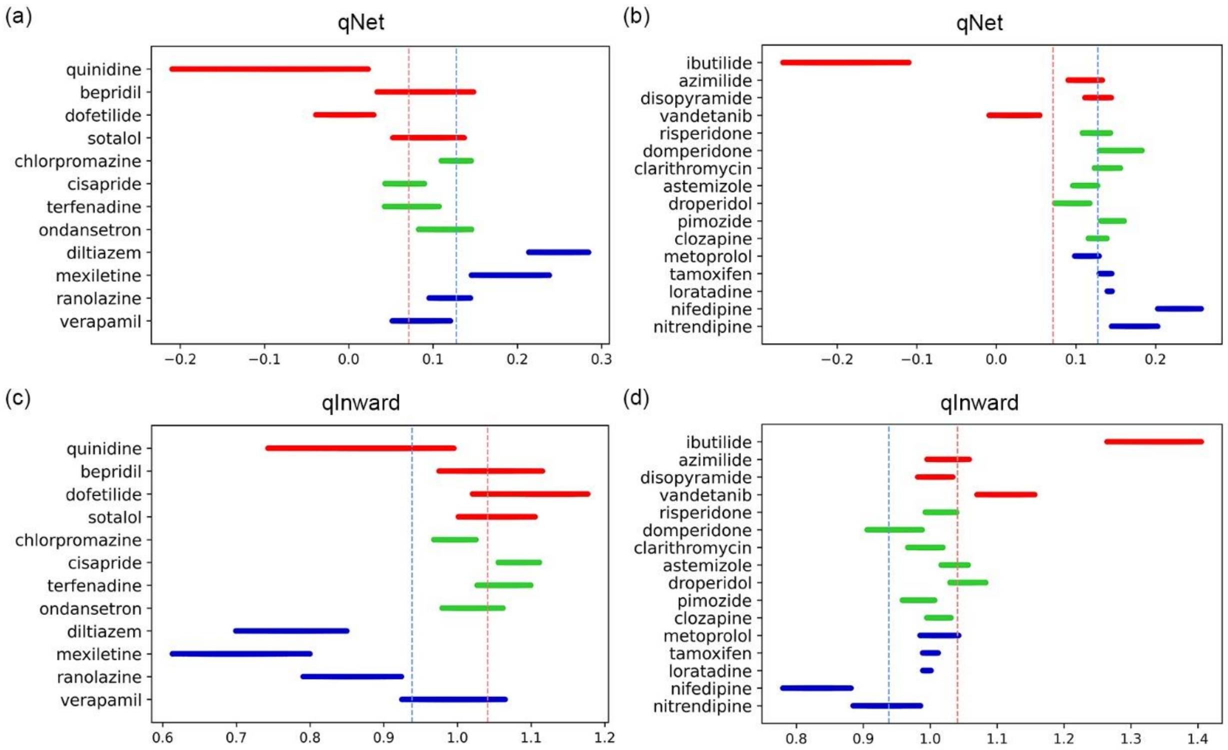

Supplementary Figure S 4. Distributions of qNet and qInward in the Li dataset; (a, c), qNet and qInward of 12 train drugs; (b, d), qNet and qInward of 16 test drugs; red, green, and blue horizontal lines denote the distribution of high, intermediate, and low-risk drugs; the blue dashed line is threshold 1 for distinguishing the low-risk drug from the high/intermediate-risk; the red dashed line is threshold 2 for determining the high-risk drug from the intermediate/low-risk.

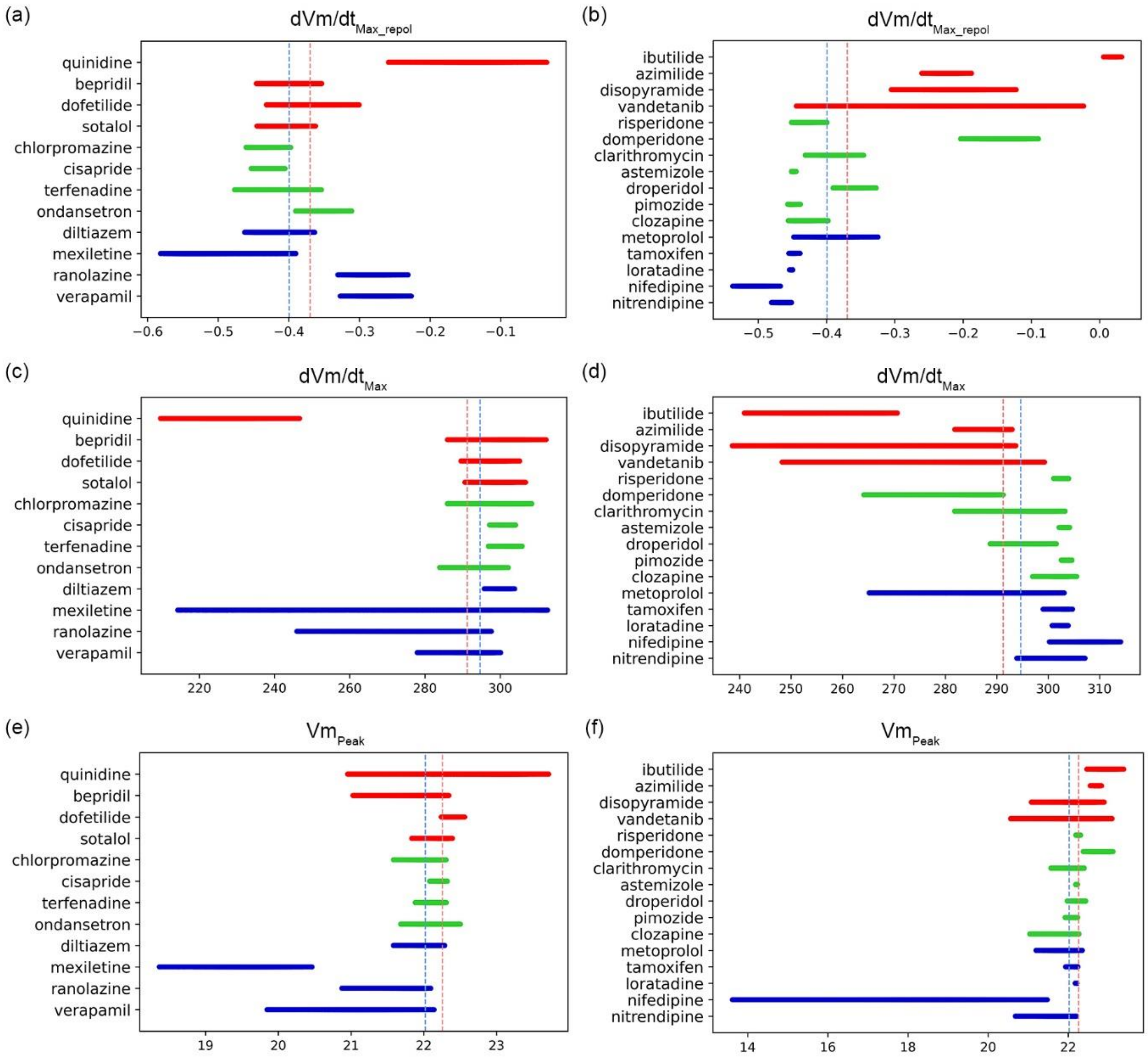

Supplementary Figure S 5. Distributions of dVm/dt_{Max_repol}, dVm/dt_{Max}, and Vm_{Peak} as *in-silico* AP features in the Chantest dataset; (a, c, e), for 12 train drugs; (b, d, f), for 16 test drugs; red, green, and blue horizontal lines denote the distribution of high, intermediate, and low-risk drugs; the blue dashed line is threshold 1 for distinguishing the low-risk drug from the high/intermediate-risk; the red dashed line is threshold 2 for determining the high-risk drug from the intermediate/low-risk.

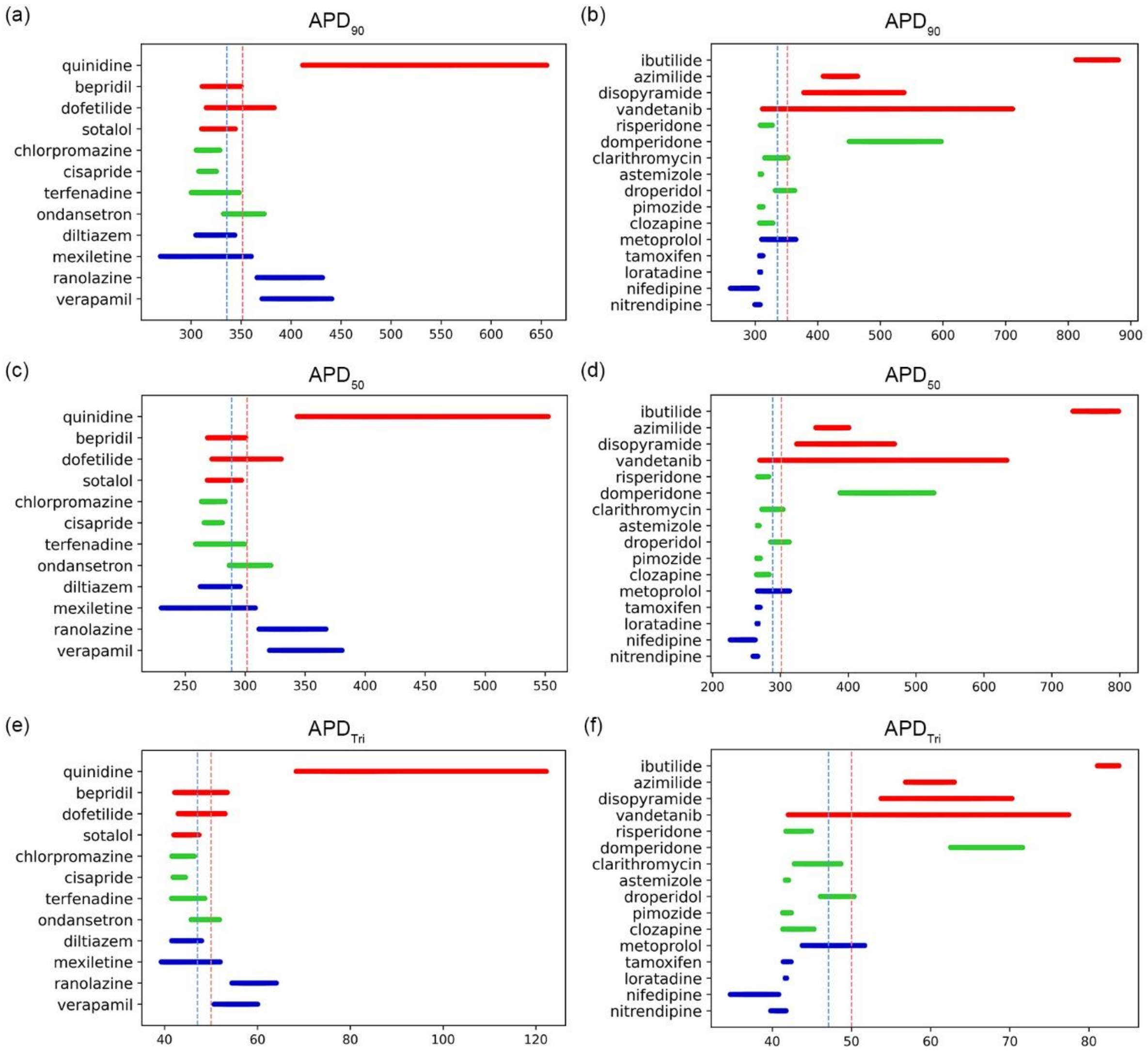

Supplementary Figure S 6. Distributions of APD_{90}, APD_{50}, and APD_{tri} as *in-silico* AP features in the Chantest dataset; (a, c, e), for 12 train drugs; (b, d, f), for 16 test drugs; red, green, and blue horizontal lines denote the distribution of high, intermediate, and low-risk drugs; the blue dashed line is threshold 1 for distinguishing the low-risk drug from the high/intermediate-risk; the red dashed line is threshold 2 for determining the high-risk drug from the intermediate/low-risk.

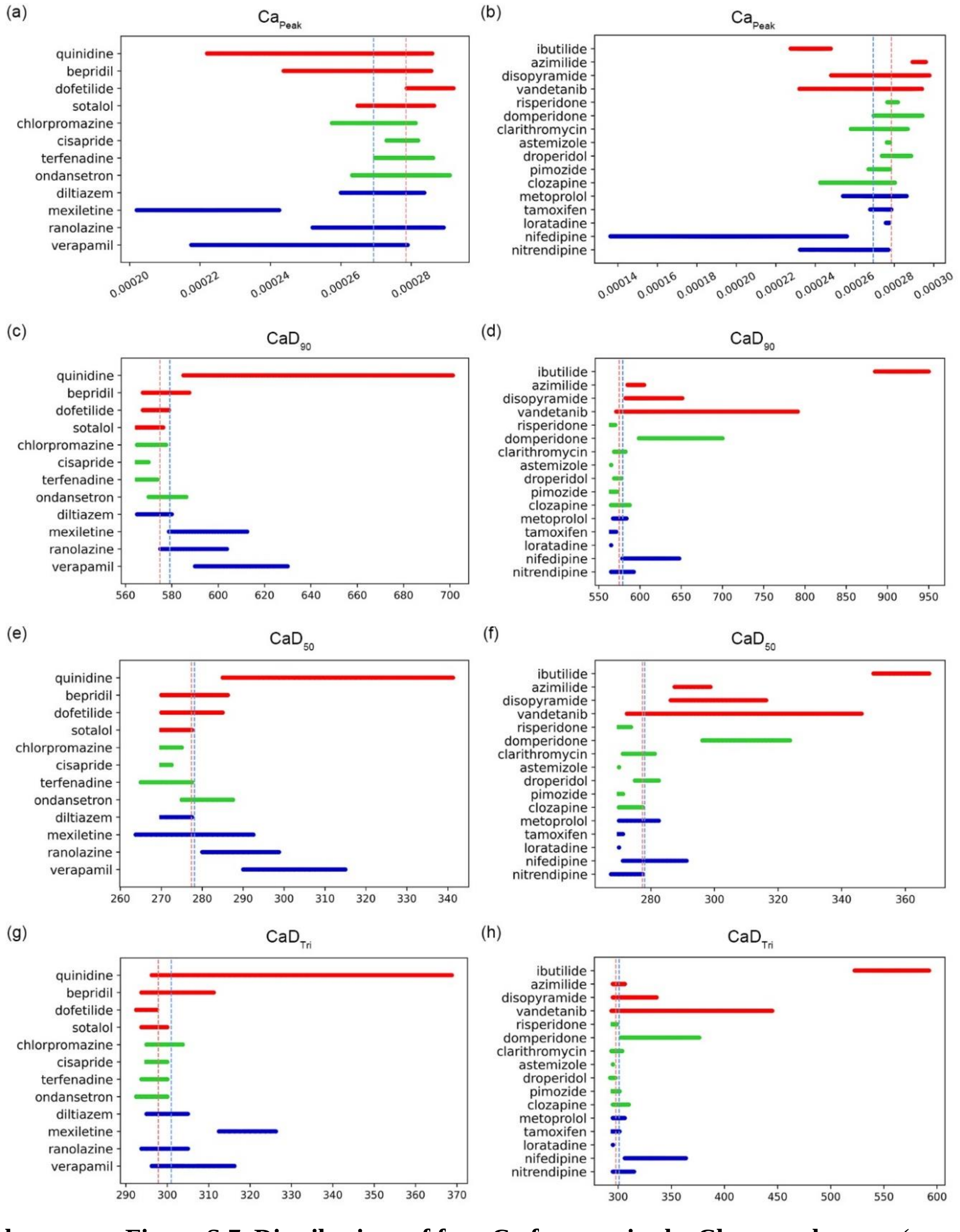

Supplementary Figure S 7. Distributions of four Ca features in the Chantest dataset; (a, c, e, g), Ca_{Peak}, CaD_{90}, CaD_{50}, and CaD_{tri} of 12 train drugs; (b, d, f, h), Ca_{Peak}, CaD_{90}, CaD_{50}, and CaD_{tri} of 16 test drugs; red, green, and blue horizontal lines denote the distribution of high, intermediate, and low-risk drugs; the blue dashed line is threshold 1 for distinguishing the low-risk drug from the high/intermediate-risk; the red dashed line is threshold 2 for determining the high-risk drug from the intermediate/low-risk.

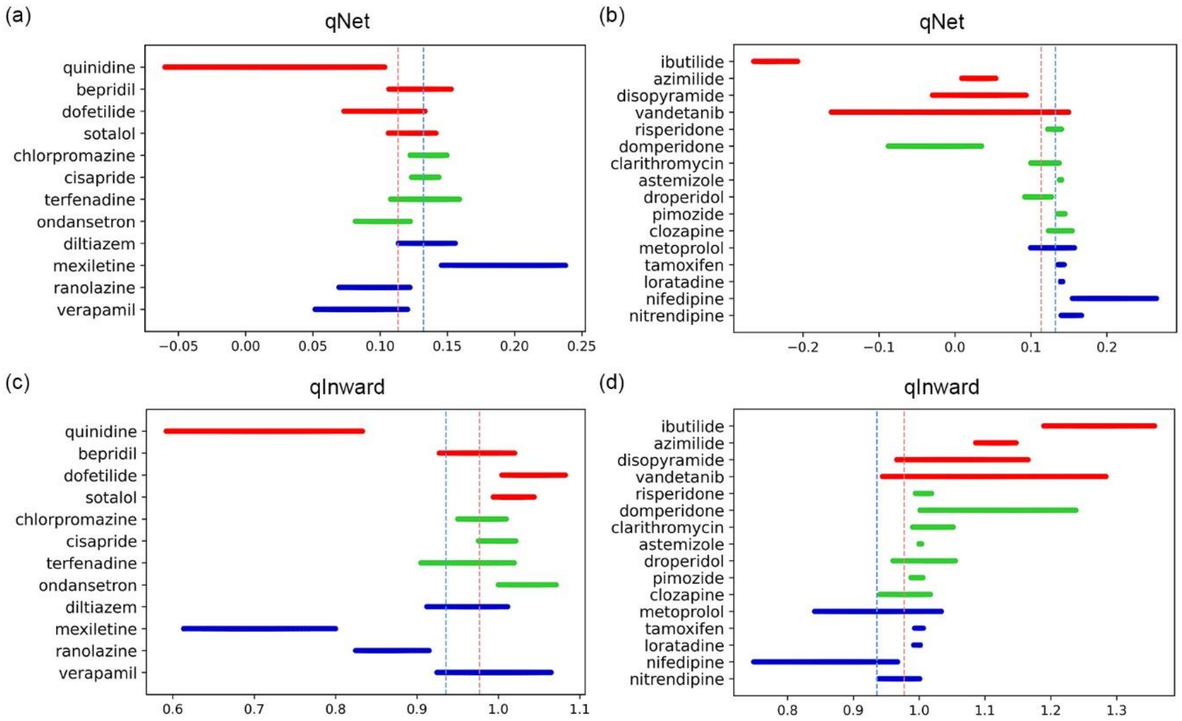

Supplementary Figure S 8. Distributions of qNet and qInward in the Chantest dataset; (a, c), qNet and qInward of 12 train drugs; (b, d), qNet and qInward of 16 test drugs; red, green, and blue horizontal lines denote the distribution of high, intermediate, and low-risk drugs; the blue dashed line is threshold 1 for distinguishing the low-risk drug from the high/intermediate-risk; the red dashed line is threshold 2 for determining the high-risk drug from the intermediate/low-risk.

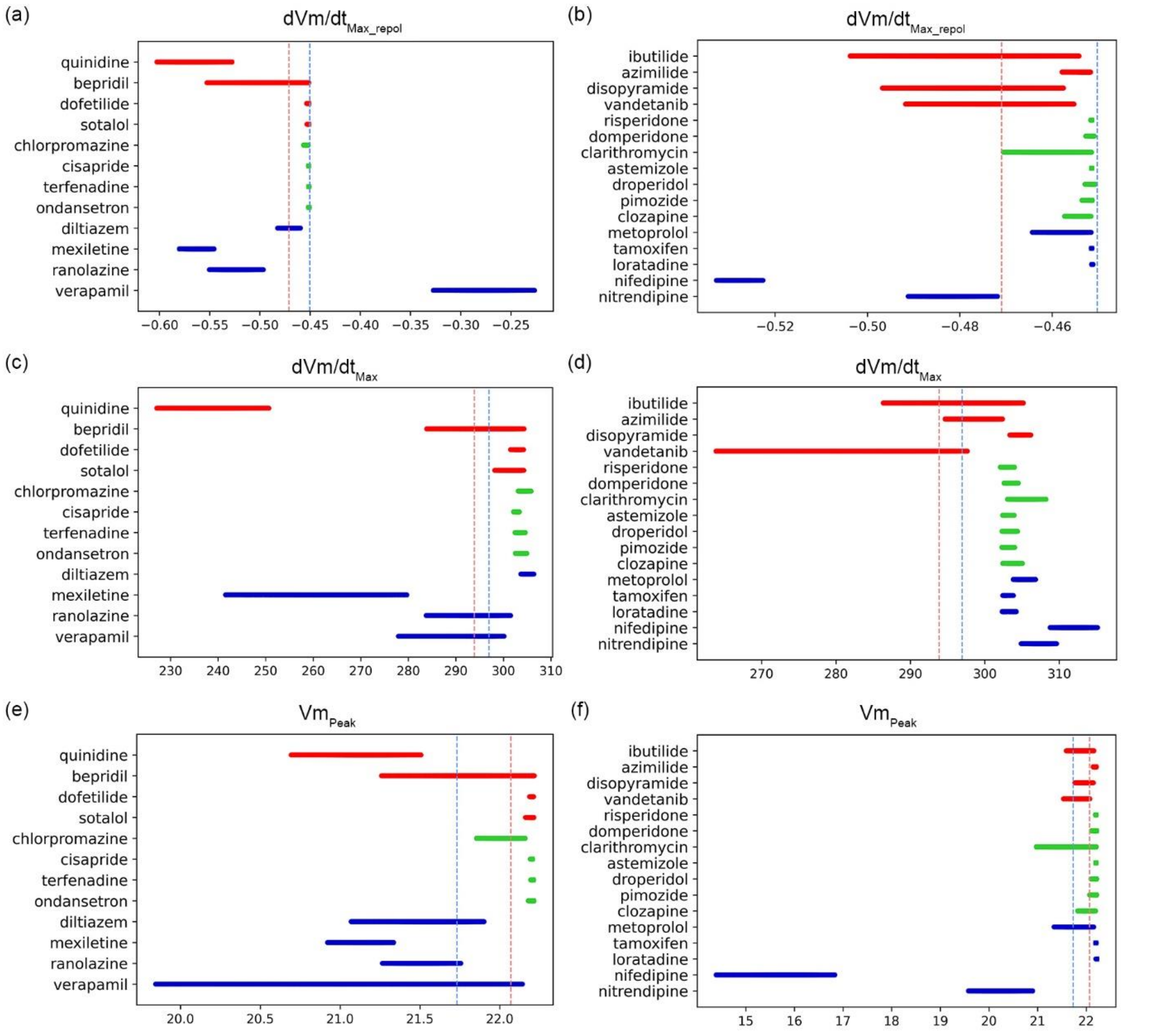

Supplementary Figure S 9. Distributions of dVm/dt_{Max_repol}, dVm/dt_{Max}, and Vm_{Peak} as *in-silico* AP features in the Nanion dataset; (a, c, e), for 12 train drugs; (b, d, f), for 16 test drugs; red, green, and blue horizontal lines denote the distribution of high, intermediate, and low-risk drugs; the blue dashed line is threshold 1 for distinguishing the low-risk drug from the high/intermediate-risk; the red dashed line is threshold 2 for determining the high-risk drug from the intermediate/low-risk.

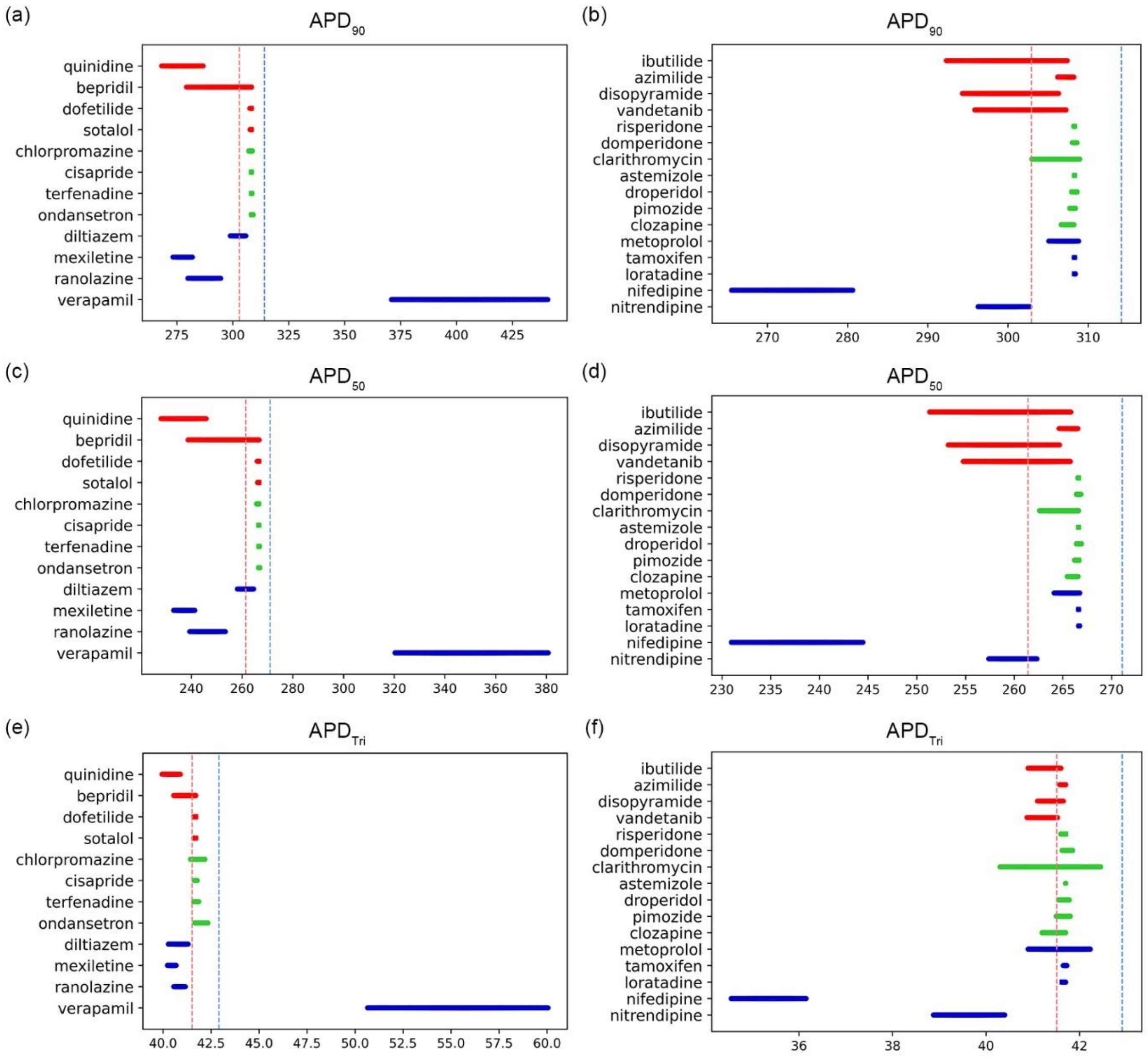

Supplementary Figure S 10. Distributions of APD_{90}, APD_{50}, and APD_{tri} as *in-silico* AP features in the Nanion dataset; (a, c, e), for 12 train drugs; (b, d, f), for 16 test drugs; red, green, and blue horizontal lines denote the distribution of high, intermediate, and low-risk drugs; the blue dashed line is threshold 1 for distinguishing the low-risk drug from the high/intermediate-risk; the red dashed line is threshold 2 for determining the high-risk drug from the intermediate/low-risk.

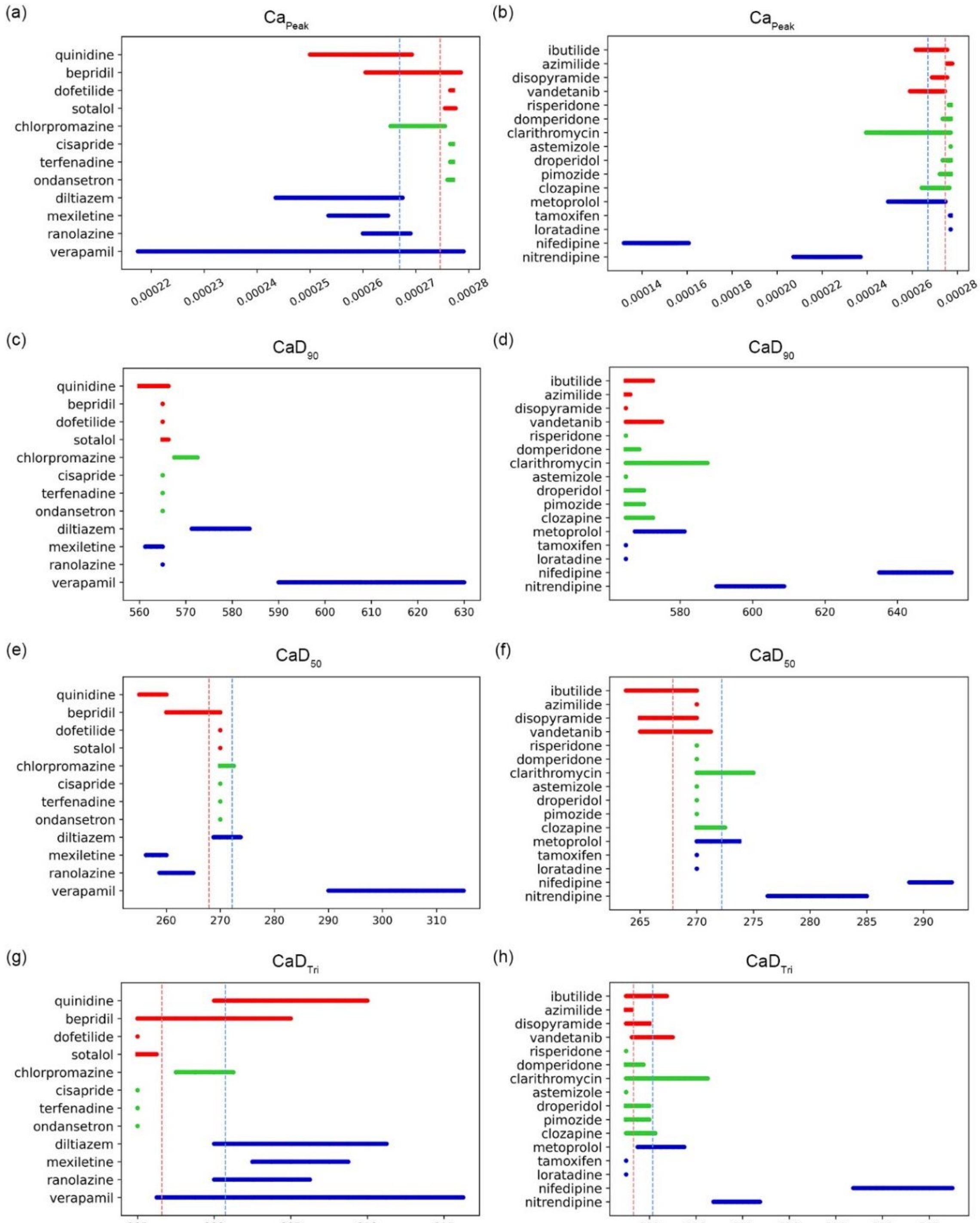

Supplementary Figure S 11. Distributions of four Ca features in the Nanion dataset; (a, c, e, g), Ca_{Peak}, CaD_{50}, and CaD_{tri} of 12 train drugs; (b, d, f, h), Ca_{Peak}, CaD_{50}, and CaD_{tri} of 16 test drugs; red, green, and blue horizontal lines denote the distribution of high, intermediate, and low-risk drugs; the blue dashed line is threshold 1 for distinguishing the low-risk drug from the high/intermediate-risk; the red dashed line is threshold 2 for determining the high-risk drug from the intermediate/low-risk.

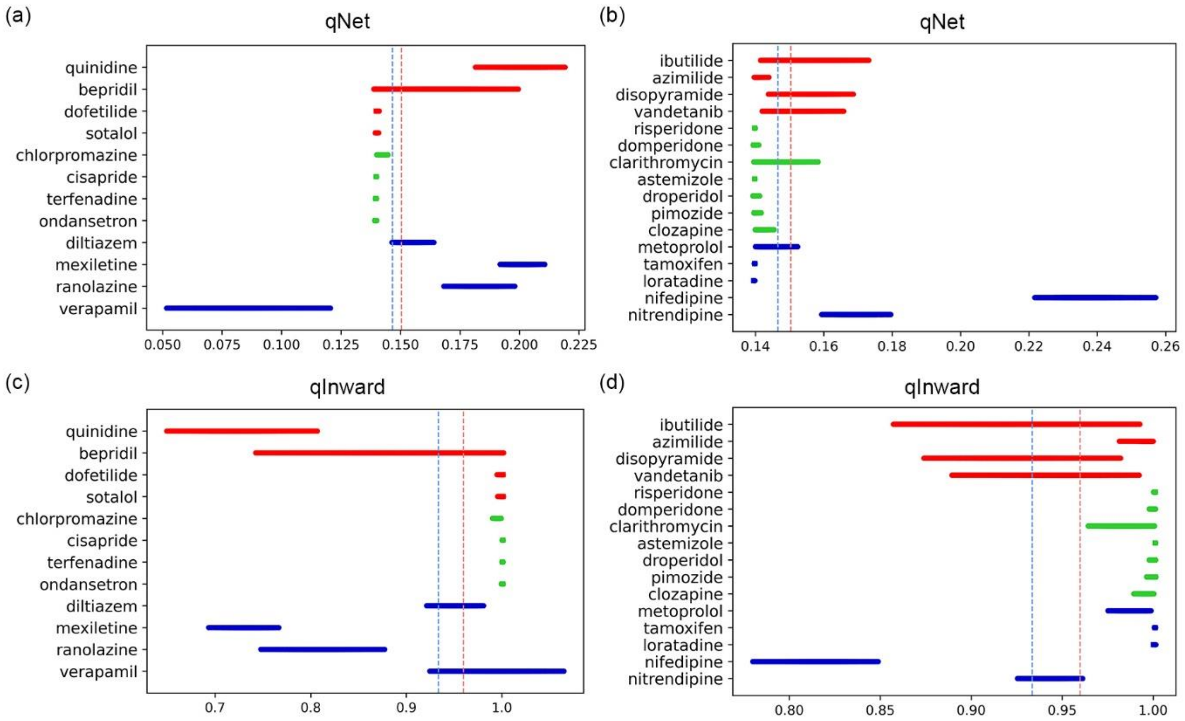

Supplementary Figure S 12. Distributions of qNet and qInward in the Nanion dataset; (a, c), qNet and qInward of 12 train drugs; (b, d), qNet and qInward of 16 test drugs; red, green, and blue horizontal lines denote the distribution of high, intermediate, and low-risk drugs; the blue dashed line is threshold 1 for distinguishing the low-risk drug from the high/intermediate-risk; the red dashed line is threshold 2 for determining the high-risk drug from the intermediate/low-risk.

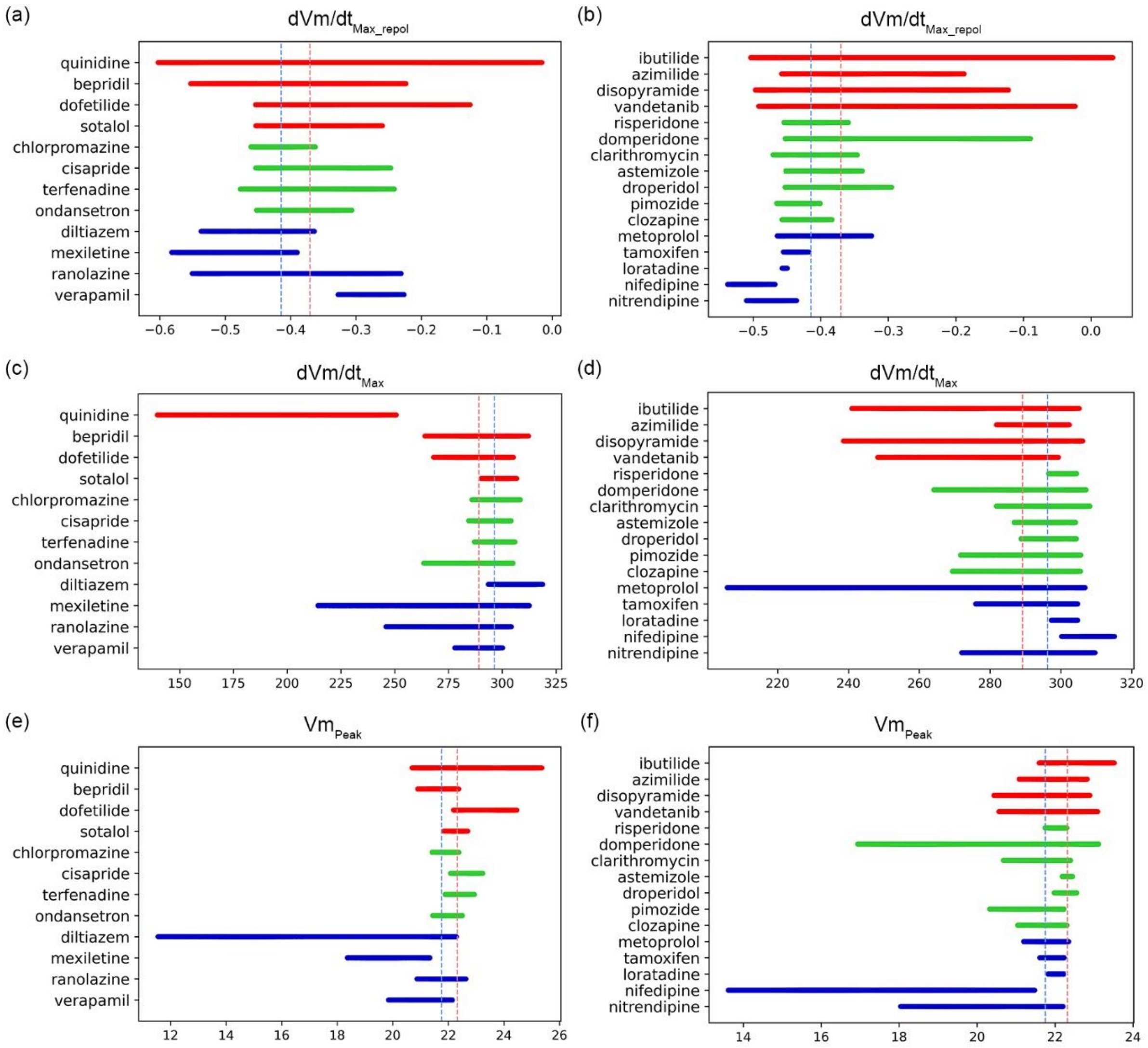

Supplementary Figure S 13. Distributions of dVm/dt_{Max_repol}, dVm/dt_{Max}, and Vm_{Peak} as *in-silico* AP features in the merge of three datasets; (a, c, e), for 12 train drugs; (b, d, f), for 16 test drugs; red, green, and blue horizontal lines denote the distribution of high, intermediate, and low-risk drugs; the blue dashed line is threshold 1 for distinguishing the low-risk drug from the high/intermediate-risk; the red dashed line is threshold 2 for determining the high-risk drug from the intermediate/low-risk.

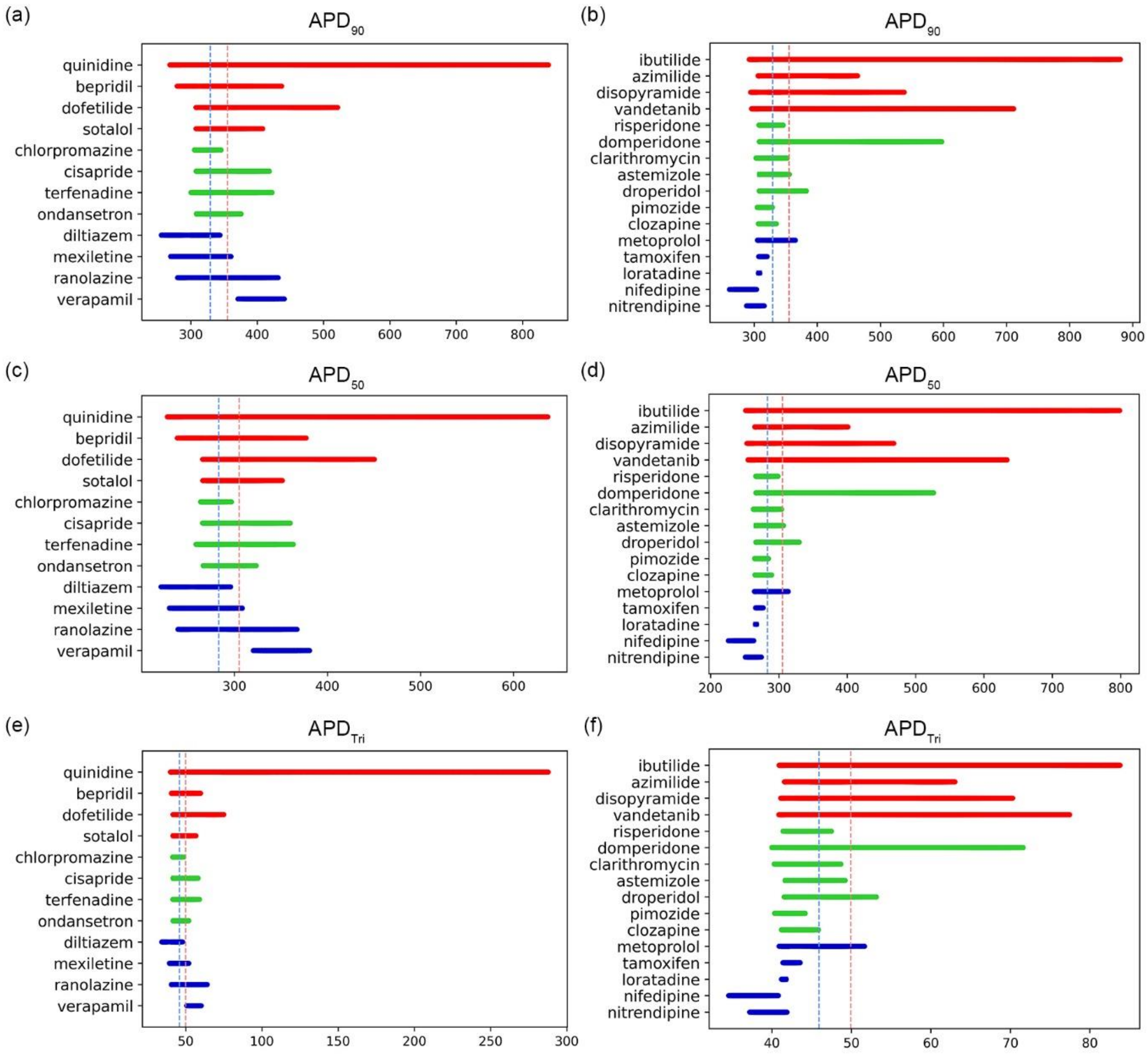

Supplementary Figure S 14. Distributions of APD_{90}, APD_{50}, and APD_{tri} as *in-silico* AP features in the merge of three datasets; (a, c, e), for 12 train drugs; (b, d, f), for 16 test drugs; red, green, and blue horizontal lines denote the distribution of high, intermediate, and low-risk drugs; the blue dashed line is threshold 1 for distinguishing the low-risk drug from the high/intermediate-risk; the red dashed line is threshold 2 for determining the high-risk drug from the intermediate/low-risk.

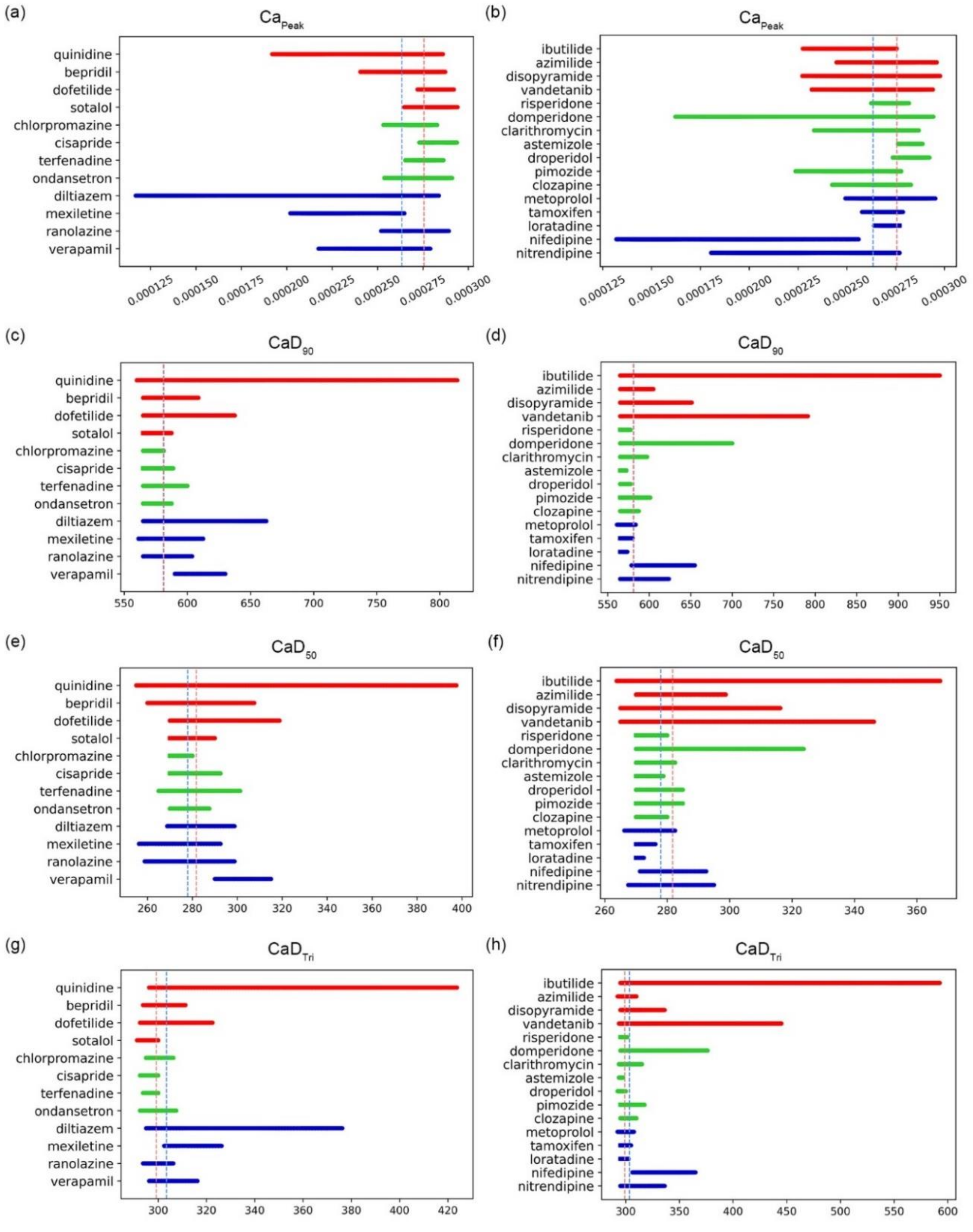

Supplementary Figure S 15. Distributions of four Ca features in the merge of three datasets; (a, c, e, g), Ca_{Peak}, CaD_{90}, CaD_{50}, and CaD_{tri} of 12 train drugs; (b, d, f, h), Ca_{Peak}, CaD_{90}, CaD_{50}, and CaD_{tri} of 16 test drugs; red, green, and blue horizontal lines denote the distribution of high, intermediate, and low-risk drugs; the blue dashed line is threshold 1 for distinguishing the low-risk drug from the high/intermediate-risk; the red dashed line is threshold 2 for determining the high-risk drug from the intermediate/low-risk.

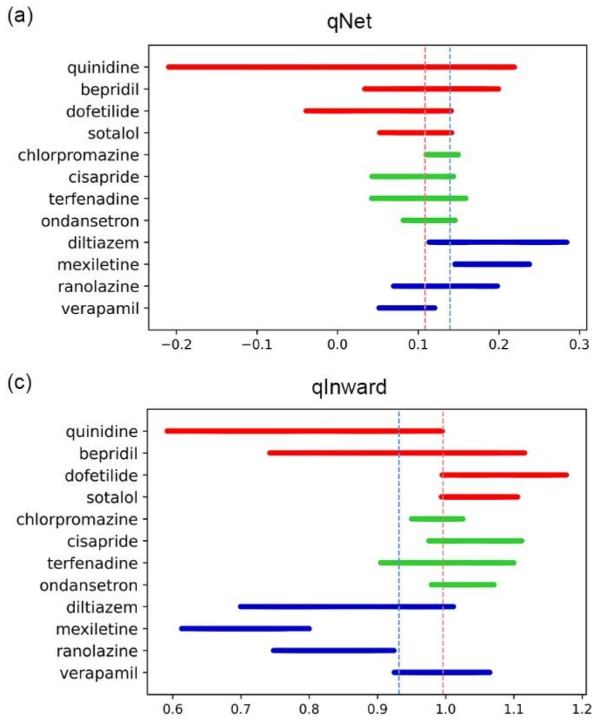

Supplementary Figure S 16. Distributions of qNet and qInward in the merge of three datasets; (a, c), qNet and qInward of 12 train drugs; (b, d), qNet and qInward of 16 test drugs; red, green, and blue horizontal lines denote the distribution of high, intermediate, and low-risk drugs; the blue dashed line is threshold 1 for distinguishing the low-risk drug from the high/intermediate-risk; the red dashed line is threshold 2 for determining the high-risk drug from the intermediate/low-risk.

1.2 Supplementary Tables

Supplementary Table S 1. Statistics of *in-silico* biomarkers according to the *in-vitro* dataset

In-silico features	*In-vitro* dataset	12-train drugs					16-test drugs				
		Mean	Variation	Min	Max	Median	Mean	Variation	Min	Max	Median
dVm/dt_{Max_Repol}	Li et al.	-0.3261	0.0153	-0.5817	-0.0158	-0.3261	-0.3773	0.0141	-0.5303	0.0205	-0.4019
	Chantest et al.	-0.3737	0.0097	-0.5817	-0.0350	-0.4064	-0.3479	0.0207	-0.5378	0.0324	-0.4198
	Nanion et al.	0.4655	0.0051	-0.6024	-0.2266	-0.4516	-0.4638	0.0004	-0.5327	-0.4507	-0.4528
dVm/dt_{Max}	Li et al.	287.2	727.8	139.7	318.7	295.4	293.6	358.8	205.8	314.5	299.2
	Chantest et al.	290.4	521.7	209.7	312.6	299.7	294.8	182.7	238.6	314.1	301.2
	Nanion et al.	292.9	393.2	227.1	306.4	302.7	303.0	24.6	263.9	315.1	303.0
Vm_{Peak}	Li et al.	21.67	6.01	11.55	25.36	22.18	21.28	2.94	14.18	23.52	21.97
	Chantest et al.	21.93	0.67	18.36	23.71	22.17	22.10	0.86	13.62	23.38	22.21
	Nanion et al.	21.80	0.20	19.85	22.22	22.03	21.56	2.41	14.39	22.22	22.16
APD_{90}	Li et al.	382.4	8,491.3	255.4	839.0	364.6	358.2	12,025.4	265.4	840.7	327.7
	Chantest et al.	350.2	3,525.8	269.5	655.6	326.1	384.3	18,849.2	260.8	880.2	320.2
	Nanion et al.	308.0	959.3	268.2	440.8	308.3	303.9	71.4	265.5	308.9	308.0
APD_{50}	Li et al.	327.7	5,729.2	221.2	636.7	313.0	311.4	10,003.0	230.9	757.0	283.2
	Chantest et al.	300.4	2,345.9	230.2	552.5	280.9	334.1	15,833.0	226.1	798.4	276.0
	Nanion et al.	265.5	735.9	228.1	380.7	266.6	262.9	48.8	231.0	266.9	266.3
APD_{tri}	Li et al.	54.7	367.3	34.2	287.7	51.9	46.8	98.7	34.55	83.65	44.12
	Chantest et al.	49.8	125.8	39.3	122.2	45.2	50.2	148.2	34.7	83.8	43.95
	Nanion et al.	42.5	16.2	40.0	60.1	41.7	41.1	2.4	34.55	42.45	41.65
Ca_{Peak}	Li et al.	0.000254	1.83E-09	0.000117	0.000294	0.000274	0.000252	1.35E-09	0.000133	0.000295	0.000266
	Chantest et al.	0.000271	3.23E-10	0.000202	0.000292	0.000278	0.000272	5.50E-10	0.000136	0.000298	0.000277
	Nanion et al.	0.000269	8.34E-11	0.000218	0.000279	0.000272	0.000262	1.07E-09	0.000132	0.000278	0.000276
CaD_{90}	Li et al.	604.2	1,810.0	567.5	813.8	587.5	604.3	3,930.4	561.2	898.8	580.0
	Chantest et al.	579.9	325.7	565.0	701.2	571.2	602.7	7,054.2	565.0	950.0	572.5
	Nanion et al.	569.3	158.7	560.0	630.0	565.0	573.9	409.4	565.0	655.0	565.0
CaD_{50}	Li et al.	294.2	677.5	263.8	397.5	287.5	286.6	431.5	266.2	360.0	277.5
	Chantest et al.	279.4	167.1	263.8	341.2	273.8	284.9	507.0	267.5	367.5	275.0
	Nanion et al.	269.9	123.8	255.0	315.0	270.0	271.7	35.6	263.8	292.5	270.0
CaD_{tri}	Li et al.	310.0	482.7	291.2	423.8	298.8	317.8	1,936.3	292.5	538.8	300.0
	Chantest et al.	300.5	63.9	292.5	368.8	297.5	317.8	4,032.7	292.5	592.5	297.5
	Nanion et al.	299.4	19.7	295.0	316.2	300.0	302.1	212.8	295.0	365.0	295.0
qNet	Li et al.	0.098	0.0064	-0.209	0.284	0.096	0.111	0.0083	-0.268	0.257	0.130
	Chantest et al.	0.120	0.0016	-0.060	0.238	0.127	0.086	0.0106	-0.265	0.265	0.134
	Nanion et al.	0.152	0.0010	0.052	0.219	0.140	0.152	0.0006	0.139	0.257	0.141
qInward	Li et al.	0.967	0.0164	0.613	1.177	1.012	1.020	0.0109	0.780	1.405	1.008
	Chantest et al.	0.940	0.0129	0.592	1.082	0.985	1.032	0.0080	0.749	1.357	1.003
	Nanion et al.	0.929	0.0113	0.649	1.065	0.997	0.971	0.0024	0.780	1.001	0.998

Supplementary Table S 2. Statistical analysis of *in silico* biomarkers according to the *in vitro* datasets.

In-silico features	Pairs of the *in-vitro* datasets	12-train drugs						16-test drugs					
		F-test			Two-samples T-test			F-test			Two-samples T-test		
		F	P value	$F_{critical}$	T-stats	P value	$T_{critical}$	F	P value	$F_{critical}$	T-stats	P value	$T_{critical}$
dVm/dt_{Max_Repol}	Li – Chantest	1.577	0.000	1.022	46.74	0.000	1.96	1.472	0.000	1.019	28.228	0.000	1.96
	Li – Nanion	3.002	0.000	1.022	151.38	0.000	1.96	34.495	0.000	1.019	128.53	0.000	1.96
	Chantest – Nanion	1.903	0.000	1.022	116.98	0.000	1.96	50.772	0.000	1.019	142.65	0.000	1.96
dVm/dt_{Max}	Li – Chantest	1.395	0.000	1.021	-13.68	0.000	1.96	1.964	0.000	1.019	-9.424	0.000	1.96
	Li – Nanion	1.851	0.000	1.021	-26.01	0.000	1.96	14.580	0.000	1.019	-85.536	0.000	1.96
	Chantest – Nanion	1.327	0.000	1.021	-12.802	0.000	1.96	7.424	0.000	1.019	-101.10	0.000	1.96
Vm_{Peak}	Li – Chantest	8.997	0.000	1.021	-15.362	0.000	1.96	1.218	0.000	1.019	-21.324	0.000	1.96
	Li – Nanion	30.733	0.000	1.021	-8.029	0.000	1.96	3.408	0.000	1.019	-74.791	0.000	1.96
	Chantest – Nanion	3.416	0.000	1.021	21.195	0.000	1.96	2.797	0.000	1.019	-53.325	0.000	1.96
APD_{90}	Li – Chantest	2.408	0.000	1.021	45.464	0.000	1.96	1.567	0.000	1.019	26.563	0.000	1.96
	Li – Nanion	8.852	0.000	1.021	118.51	0.000	1.96	168.31	0.000	1.019	88.266	0.000	1.96
	Chantest – Nanion	3.676	0.000	1.021	97.607	0.000	1.96	263.82	0.000	1.019	104.51	0.000	1.96
APD_{50}	Li – Chantest	2.442	0.000	1.021	47.069	0.000	1.96	1.583	0.000	1.019	25.238	0.000	1.96
	Li – Nanion	7.786	0.000	1.021	119.86	0.000	1.96	205.07	0.000	1.019	86.530	0.000	1.96
	Chantest – Nanion	3.188	0.000	1.021	97.412	0.000	1.96	324.59	0.000	1.019	101.03	0.000	1.96
APD_{tri}	Li – Chantest	2.920	0.000	1.021	33.964	0.000	1.96	1.503	0.000	1.019	38.878	0.000	1.96
	Li – Nanion	22.673	0.000	1.021	96.170	0.000	1.96	41.026	0.000	1.019	102.72	0.000	1.96
	Chantest – Nanion	7.766	0.000	1.021	94.750	0.000	1.96	61.647	0.000	1.019	133.90	0.000	1.96
Ca_{Peak}	Li – Chantest	5.664	0.000	1.021	-58.199	0.000	1.96	2.459	0.000	1.019	-81.654	0.000	1.96
	Li – Nanion	21.972	0.000	1.021	-52.949	0.000	1.96	1.265	0.000	1.019	-35.961	0.000	1.96
	Chantest – Nanion	3.879	0.000	1.021	19.072	0.000	1.96	1.944	0.000	1.019	-44.540	0.000	1.96
CaD_{90}	Li – Chantest	5.557	0.000	1.021	81.417	0.000	1.96	1.795	0.000	1.019	-2.773	0.000	1.96
	Li – Nanion	11.406	0.000	1.021	121.95	0.000	1.96	9.600	0.000	1.019	82.668	0.000	1.96
	Chantest – Nanion	2.053	0.000	1.021	74.900	0.000	1.96	17.230	0.000	1.019	59.673	0.000	1.96
CaD_{50}	Li – Chantest	4.055	0.000	1.021	78.860	0.000	1.96	1.175	0.000	1.019	-9.685	0.000	1.96
	Li – Nanion	5.473	0.000	1.021	133.39	0.000	1.96	12.113	0.000	1.019	122.61	0.000	1.96
	Chantest – Nanion	1.350	0.000	1.021	87.021	0.000	1.96	14.231	0.000	1.019	101.04	0.000	1.96
CaD_{tri}	Li – Chantest	7.557	0.000	1.021	62.907	0.000	1.96	2.083	0.000	1.019	0.078	0.000	1.96
	Li – Nanion	24.455	0.000	1.021	72.943	0.000	1.96	9.101	0.000	1.019	60.309	0.000	1.96
	Chantest – Nanion	3.236	0.000	1.021	17.973	0.000	1.96	18.954	0.000	1.019	43.001	0.000	1.96
qNet	Li – Chantest	3.984	0.000	1.021	-38.393	0.000	1.96	1.281	0.000	1.019	-32.521	0.000	1.96
	Li – Nanion	6.434	0.000	1.021	-96.930	0.000	1.96	14.544	0.000	1.019	-78.523	0.000	1.96
	Chantest – Nanion	1.615	0.000	1.021	-96.072	0.000	1.96	18.633	0.000	1.019	-112.14	0.000	1.96
qInward	Li – Chantest	1.272	0.000	1.021	24.364	0.000	1.96	1.362	0.000	1.019	-15.167	0.000	1.96
	Li – Nanion	1.445	0.000	1.021	35.868	0.000	1.96	4.522	0.000	1.019	76.660	0.000	1.96
	Chantest – Nanion	1.136	0.000	1.021	11.594	0.000	1.96	3.321	0.000	1.019	107.09	0.000	1.96

Supplementary Table S 3. Likelihood ratios of the logistic regression using action potential (AP) features; likelihood ratios according to the AP features show the median, minimal and maximal values after evaluating the model through the 10,000-test algorithm, Merged, the combined set containing all three datasets.; LR+, positive likelihood ratio; LR-, negative likelihood ratio; LR+>2 = Minimal, LR+>5 = Good, *LR+>10 = Excellent; LR-<0.5 = Minimal, LR-<0.2 = Good, *LR-<0.1 = Excellent.

AP feature	Dataset	LR+			LR-		
		Low	Inter	High	Low	Inter	High
dVm/dt_{Max_Repol}	Li.	1.57 (1.10 – 2.93)	1.29 (0.00 – inf)	*inf (inf – inf)	0.37 (0.00 – 0.73)	0.92 (0.48 – 1.50)	0.50 (0.50 – 0.50)
	Chantest	2.20 (1.47 – 3.67)	1.29 (0.00 – inf)	6.00 (3.00 – 12.00)	0.31 (0.00 – 0.44)	0.96 (0.64 – 1.29)	*0.00 (0.00 – 0.33)
	Nanion	1.00 (1.00 – 1.00)	1.80 (1.29 – 2.25)	3.00 (0.00 – 4.50)	1.00 (1.00 – 1.00)	*0.00 (0.00 – 0.00)	0.60 (0.30 – 1.20)
	Merged	2.93 (1.76 – inf)	1.93 (0.00 – inf)	6.00 (1.50 – inf)	0.28 (0.00 – 0.37)	0.73 (0.16 – 1.50)	0.30 (0.00 – 0.75)
dVm/dt_{Max}	Li.	1.26 (0.49 – 2.93)	1.29 (0.00 – inf)	3.00 (1.00 – 9.00)	0.55 (0.28 – 3.30)	0.96 (0.32 – 1.80)	0.82 (0.27 – 1.00)
	Chantest	1.47 (1.10 – 2.20)	0.00 (0.00 – inf)	6.00 (1.00 – inf)	0.44 (0.00 – 0.88)	1.12 (0.80 – 1.80)	0.27 (0.00 – 1.00)
	Nanion	1.10 (1.00 – 1.37)	1.00 (0.00 – 1.00)	*inf (1.00 – inf)	*0.00 (0.00 – 1.00)	1.00 (1.00 – 1.50)	0.75 (0.50 – 1.00)
	Merged	1.10 (0.49 – 2.20)	0.86 (0.00 – inf)	3.00 (0.75 – 9.00)	0.73 (0.31 – 3.30)	1.07 (0.55 – 1.80)	0.82 (0.27 – 1.12)
Vm_{Peak}	Li.	1.10 (0.63 – 4.40)	1.29 (0.77 – 2.57)	*Inf (inf – inf)	0.94 (0.49 – 1.65)	0.77 (0.21 – 1.29)	0.75 (0.75 – 0.75)
	Chantest	*inf (0.55 – inf)	1.71 (0.32 – 6.43)	4.00 (1.20 – 12.00)	0.60 (0.20 – 1.26)	0.64 (0.18 – 1.71)	0.30 (0.00 – 0.86)
	Nanion	4.40 (1.47 – inf)	0.32 (0.00 – 2.57)	0.38 (0.30 – 1.12)	0.66 (0.40 – 0.83)	1.54 (0.80 – 1.80)	2.25 (0.75 – 4.50)
	Merged	0.88 (0.63 – 3.30)	0.64 (0.26 – 1.71)	1.50 (1.00 – inf)	1.10 (0.49 – 1.65)	1.29 (0.51 – 2.14)	0.90 (0.55 – 1.00)
APD_{90}	Li.	1.57 (0.98 – 2.75)	2.57 (0.00 – inf)	*inf (inf – inf)	*0.00 (0.00 – 1.10)	0.86 (0.57 – 1.50)	0.50 (0.50 – 0.50)
	Chantest	2.20 (1.47 – 2.75)	*inf (0.00 – inf)	*12.0 (4.0 – 12.0)	*0.00 (0.00 – 0.44)	0.86 (0.71 – 1.29)	*0.00 (0.00 – 0.30)
	Nanion	1.00 (1.00 – 1.00)	1.80 (1.29 – 2.25)	3.00 (0.00 – 4.50)	1.00 (1.00 – 1.00)	*0.00 (0.00 – 0.00)	0.60 (0.30 – 1.20)
	Merged	2.20 (1.26 – 4.40)	1.29 (0.00 – 5.14)	*inf (3.00 – inf)	0.31 (0.00 – 0.55)	0.86 (0.37 – 1.50)	0.50 (0.25 – 0.60)
APD_{50}	Li.	1.57 (0.98 – 2.75)	2.57 (0.00 – inf)	*inf (inf – inf)	*0.00 (0.00 – 1.10)	0.80 (0.43 – 1.29)	0.50 (0.50 – 0.50)
	Chantest	2.20 (1.47 – 2.75)	*inf (0.00 – inf)	*12.0 (4.0 – 12.0)	*0.00 (0.00 – 0.44)	0.86 (0.71 – 1.29)	*0.00 (0.00 – 0.30)
	Nanion	1.00 (1.00 – 1.00)	1.80 (1.12 – 2.25)	3.00 (0.00 – 9.00)	1.00 (1.00 – 1.00)	*0.00 (0.00 – 0.00)	0.60 (0.27 – 1.20)
	Merged	2.20 (1.47 – 4.40)	1.29 (0.00 – inf)	*inf (3.00 – inf)	0.31 (0.00 – 0.44)	0.86 (0.32 – 1.50)	0.50 (0.25 – 0.60)
APD_{tri}	Li.	1.37 (0.98 – 1.83)	*inf (0.00 – inf)	*Inf (inf – inf)	*0.00 (0.00 – 1.10)	0.86 (0.71 – 1.29)	0.50 (0.50 – 0.50)
	Chantest	1.76 (1.26 – 2.75)	1.29 (0.00 – inf)	*12.0 (4.5 – 12.0)	0.37 (0.00 – 0.55)	0.96 (0.71 – 1.29)	*0.00 (0.00 – 0.30)
	Nanion	1.00 (1.00 – 1.00)	1.93 (1.07 – 3.00)	3.00 (0.75 – 4.50)	1.00 (1.00 – 1.00)	0.26 (0.00 – 0.86)	0.33 (0.30 – 1.12)
	Merged	1.47 (0.98 – 2.75)	1.29 (0.00 – inf)	*inf (6.00 – inf)	0.44 (0.00 – 1.10)	0.92 (0.57 – 1.50)	0.50 (0.25 – 0.55)

Supplementary Table S 4. Likelihood ratios of the logistic regression using calcium (Ca) features; likelihood ratios according to the AP features show the median, minimal and maximal values after evaluating the model through the 10,000-test algorithm, Merged, the combined set containing all three datasets; LR+, positive likelihood ratio; LR-, negative likelihood ratio; LR+>2 = Minimal, LR+>5 = Good, *LR+>10 = Excellent; LR-<0.5 = Minimal, LR-<0.2 = Good, *LR-<0.1 = Excellent

Ca feature	Dataset	LR+			LR-		
		Low	Inter	High	Low	Inter	High
Ca Peak	Li.	2.20 (0.44 – 4.40)	0.64 (0.00 – inf)	0.60 (0.00 – 2.00)	0.73 (0.66 – 1.47)	1.29 (0.48 – 3.21)	1.29 (0.67 – 3.00)
	Chantest	4.40 (0.55 – 6.60)	1.29 (0.32 – 3.86)	2.25 (0.50 – 9.00)	0.66 (0.44 – 1.26)	0.77 (0.18 – 1.71)	0.38 (0.27 – 1.50)
	Nanion	6.60 (1.47 – inf)	0.43 (0.00 – 2.57)	0.43 (0.33 – 1.29)	0.44 (0.40 – 0.83)	1.29 (0.80 – 1.80)	1.80 (0.60 – 3.00)
	Merged	0.73 (0.55 – 1.47)	0.86 (0.00 – inf)	0.00 (0.00 – 3.00)	1.32 (0.73 – 2.20)	1.07 (0.64 – 1.93)	1.33 (0.60 – 2.00)
CaD90	Li.	0.83 (0.83 – 1.26)	1.00 (0.00 – inf)	2.00 (2.00 – 4.50)	1.47 (0.55 – 1.47)	1.00 (0.86 – 1.29)	0.67 (0.30 – 0.67)
	Chantest	0.44 (0.00 – 1.65)	1.00 (0.00 – inf)	0.00 (0.00 – 0.38)	1.47 (0.63 – 1.83)	1.00 (0.64 – 1.50)	4.00 (2.00 – 6.00)
	Nanion	-	-	-	-	-	-
	Merged	0.73 (0.31 – 1.47)	1.00 (0.00 – inf)	0.43 (0.00 – 0.86)	1.32 (0.83 – 2.20)	1.00 (0.57 – 1.29)	1.80 (1.20 – 4.00)
CaD50	Li.	0.83 (0.83 – 1.47)	0.00 (0.00 – 1.29)	6.00 (6.00 – 6.00)	1.47 (0.44 – 1.47)	1.29 (0.96 – 1.80)	0.55 (0.55 – 0.55)
	Chantest	0.44 (0.00 – 1.10)	1.00 (0.00 – inf)	0.00 (0.00 – 0.43)	1.47 (0.94 – 2.75)	1.00 (0.71 – 1.29)	4.00 (1.80 – 12.00)
	Nanion	6.60 (2.20 – inf)	1.80 (1.07 – 3.00)	*inf (1.00 – inf)	0.44 (0.40 – 0.73)	0.26 (0.00 – 0.86)	0.50 (0.25 – 1.00)
	Merged	1.32 (0.63 – 3.30)	1.29 (0.00 – inf)	3.00 (1.50 – 6.00)	0.73 (0.28 – 1.65)	0.96 (0.43 – 1.50)	0.33 (0.00 – 0.75)
Catri	Li.	1.47 (0.44 – 2.20)	1.29 (0.00 – inf)	0.43 (0.00 – 1.50)	0.83 (0.73 – 1.47)	0.96 (0.64 – 1.50)	1.80 (0.50 – 4.00)
	Chantest	0.88 (0.37 – 3.30)	0.00 (0.00 – inf)	0.00 (0.00 – 1.29)	1.10 (0.49 – 1.76)	1.12 (0.57 – 2.25)	3.00 (0.60 – 6.00)
	Nanion	4.40 (1.10 – inf)	0.43 (0.00 – 2.57)	0.43 (0.33 – 1.29)	0.49 (0.40 – 0.94)	1.29 (0.73 – 1.80)	1.80 (0.60 – 3.00)
	Merged	0.88 (0.63 – 1.65)	0.43 (0.00 – inf)	0.60 (0.00 – 2.00)	1.10 (0.63 – 1.65)	1.12 (0.57 – 2.25)	1.29 (0.67 – 2.40)

Supplementary Table S 5. Likelihood ratios of the logistic regression using ion charge features; likelihood ratios according to the AP features show the median, minimal and maximal values after evaluating the model through the 10,000-test algorithm; Merged, the combined set containing all three datasets; LR+, positive likelihood ratio; LR-, negative likelihood ratio; LR+>2 = Minimal, LR+>5 = Good, *LR+>10 = Excellent; LR-<0.5 = Minimal, LR-<0.2 = Good, *LR-<0.1 = Excellent.

Ion charge feature	Dataset	LR+			LR-		
		Low	Inter	High	Low	Inter	High
qNet	Li.	1.76 (1.26 – 4.40)	1.29 (0.86 – 5.14)	*inf (inf – inf)	0.37 (0.00 – 0.55)	0.86 (0.37 – 1.07)	0.50 (0.50 – 0.50)
	Chantest	2.75 (1.76 – 5.50)	1.29 (0.00 – inf)	6.00 (2.25 – 12.00)	0.28 (0.00 – 0.37)	0.96 (0.43 – 1.29)	*0.00 (0.00 – 0.38)
	Nanion	0.83 (0.49 – 0.94)	1.00 (0.00 – inf)	3.00 (0.00 – 4.50)	1.47 (1.10 – 4.40)	1.00 (0.86 – 1.50)	0.33 (0.30 – 1.33)
	Merged	3.30 (1.65 – inf)	1.29 (0.32 – inf)	6.00 (2.00 – inf)	0.49 (0.20 – 0.73)	0.77 (0.00 – 1.54)	0.55 (0.25 – 0.67)
qInward	Li.	*inf (2.20 – inf)	1.54 (0.86 – 2.25)	6.00 (2.00 – inf)	0.60 (0.60 – 0.88)	0.32 (0.00 – 1.29)	0.55 (0.25 – 0.67)
	Chantest	*inf (1.00 – inf)	0.00 (0.00 – inf)	1.33 (0.90 – 1.71)	0.60 (0.60 – 1.00)	1.12 (0.71 – 1.50)	*0.00 (0.00 – 1.50)
	Nanion	1.10 (0.73 – inf)	0.00 (0.00 – 1.00)	0.30 (0.27 – 1.20)	0.98 (0.60 – 1.10)	1.29 (1.00 – 1.80)	4.50 (0.00 – 9.00)
	Merged	*inf (2.20 – inf)	2.57 (0.00 – inf)	1.71 (1.00 – 3.00)	0.66 (0.60 – 0.88)	0.80 (0.43 – 1.80)	*0.00 (0.00 – 1.00)
